# Dose-optimised recombinant human thrombopoietin *versus* eltrombopag in patients with immune thrombocytopenia: a multicenter, randomised controlled trial (The TE-ITP Study)

**DOI:** 10.1016/j.eclinm.2025.103459

**Published:** 2025-08-21

**Authors:** Yunfei Chen, Ting Sun, Da Gao, Wei Wang, Zeping Zhou, Guangxun Gao, Yi Wang, Hu Zhou, Yanping Song, Yinghui Lai, Zhenyu Yan, Jinsong Yan, Jie Bai, Lei Zhang

**Affiliations:** a State Key Laboratory of Experimental Hematology, National Clinical Research Center for Blood Diseases, Haihe Laboratory of Cell Ecosystem, Tianjin Key Laboratory of Gene Therapy for Blood Diseases, CAMS Key Laboratory of Gene Therapy for Blood Diseases, Institute of Hematology & Blood Diseases Hospital, Chinese Academy of Medical Sciences & Peking Union Medical College, Tianjin, China; b Department of Hematology, The Affiliated Hospital of Inner Mongolia Medical University, Hohhot, China; c Department of Hematology, The Second Affiliated Hospital of Harbin Medical University, Harbin, China; d Department of Hematology, The Second Affiliated Hospital of Kunming Medical University, Kunming, China; e Department of Hematology, The First Affiliated Hospital of Airforce Medical University, Xi'an, China; f Department of Hematology, Shaanxi Provincial People's Hospital, Xi'an, China; g Department of Hematology, Affiliated Cancer Hospital of Zhengzhou University, Henan Cancer Hospital, Zhengzhou, China; h Institute of Hematology, Central Hospital of Xi'an, Xian, China; i Department of Hematology, The Second Affiliated Hospital of Guangxi Medical University, Nanning, China; j Department of Hematology, North China University of Science and Technology Affiliated Hospital, Tangshan, China; k Department of Hematology, The Second Hospital of Dalian Medical University, Dalian, China; l Department of Hematology, The Second Hospital of Tianjin Medical University, Tianjin, China; m Tianjin Institutes of Health Science, Tianjin, China; n School of Population Medicine and Public Health, Chinese Academy of Medical Sciences and Peking Union Medical College, Beijing, China

**Keywords:** Immune thrombocytopenia, Recombinant human thrombopoietin, Eltrombopag, Individualized dosing, Platelet count

## Abstract

**Background:**

Recombinant human thrombopoietin (rhTPO) at a fixed dose of 300 U/kg/day for 2 weeks has demonstrated good efficacy and safety in adults with immune thrombocytopenia (ITP). This trial aimed to develop a flexible and personalized rhTPO regimen that ensures efficacy and safety beyond previous fixed dose, with eltrombopag as an active comparator.

**Methods:**

The TE-ITP trial was conducted in 12 centers across China. Adult ITP patients with platelet count <30 × 10^9^/L were randomised (2:1) to receive rhTPO or eltrombopag. The initial dose in patients with baseline platelet count of 20–30 × 10^9^/L *versus* <20 × 10^9^/L was 300 *versus* 600 U/kg/day for rhTPO and 25 *versus* 50 mg/day for eltrombopag, respectively. Dosage was adjusted weekly according to platelet count, with maximum of 600 U/kg/day for rhTPO and 75 mg/day for eltrombopag. The primary endpoint was the time to first platelet count ≥50 × 10^9^/L. The trial is registered on ClinicalTrials.gov (NCT05583838).

**Findings:**

Between November 22, 2022 and January 16, 2024, the trial enrolled 157 patients (median age: 52 years; 104 women): 105 and 52 in the rhTPO and eltrombopag groups, respectively. Baseline platelet count was <20 × 10^9^/L in 57.1% (60/105) and 57.7% (30/52) in the rhTPO and eltrombopag groups, respectively. The median time to the first platelet count ≥50 × 10^9^/L was 7 days (95% CI 6.0–7.0) in the rhTPO group *versus* 15 days (95% CI 9.0–25.0) in the eltrombopag group (*p* < 0.001). The risk of bleeding was lower in the rhTPO group (OR 0.523, 95% CI 0.360–0.758; *p* < 0.001). Adverse events occurred in 45.7% (48/105) and 60.8% (31/52) in the rhTPO and eltrombopag groups, respectively.

**Interpretation:**

The optimised rhTPO regimen, with individualized dosing based on platelet response, showed faster platelet elevation and lower bleeding risk than eltrombopag.

**Funding:**

This trial was supported by grants from the CAMS Innovation Fund for Medical Sciences (CIFMS) (2023-I2M-2-007), Noncommunicable Chronic Diseases-10.13039/501100018537National Science and Technology Major Project (2023ZD0500803), 10.13039/501100001809National Natural Science Foundation of China (82430010), 10.13039/501100010041Tianjin Municipal Science and Technology Commission Grant (24ZXZSSS00230).

**Research in context**

**Evidence before this study**

We searched PubMed, Web of Science, Embase for publications from database inception using the terms [(“recombinant human thrombopoietin” OR “rhTPO”) AND “eltrombopag” AND (“immune thrombocytopenia” OR “ITP”)]and identified three relevant studies comparing rhTPO and eltrombopag in adult patients with immune thrombocytopenia (ITP). One was a randomised controlled trial (RCT) comparing fixed-dose rhTPO (300 U/kg/day) versus eltrombopag (25 mg/day), which demonstrated superior efficacy and a faster platelet response with rhTPO, but the treatment duration was limited to only two weeks, and no dose optimisation strategy was employed. The second study was an observational study evaluating the safety and efficacy of switching between rhTPO and eltrombopag in patients who had an inadequate response or intolerance to their initial TPO receptor agonist (TPO-RA) therapy. The third study was one open-label, randomised, phase 2 trial with small sample size (n = 60), which showed superior sustained responses at 6 months of eltrombopag plus rhTPO compared to eltrombopag as treatment for corticosteroid-resistant or relapsed ITP patients during the COVID-19 pandemic. In summary, there are rare high-quality randomised controlled trials directly comparing rhTPO and eltrombopag, particularly those employing individualized dose optimisation strategies.

**Added value of this study**

This randomised controlled trial evaluated the efficacy of individualized rhTPO and eltrombopag dosing regimens in ITP patients. Initial doses were 300 U/kg/day of rhTPO and 25 mg/day of eltrombopag for patients with a baseline platelet count of 20–30 × 10^9^/L, while those with a baseline platelet count below 20 × 10^9^/L received 600 U/kg/day of rhTPO and 50 mg/day of eltrombopag. Comparative analysis revealed that rhTPO optimised regimen demonstrated superior clinical efficacy, achieving shorter time to the first platelet count ≥50 × 10^9^/L and reduced bleeding risk compared to eltrombopag therapy.

**Implications of all the available evidence**

These findings support the use of this tailored rhTPO regimen in ITP patients, especially in patients at a high-risk of bleeding who require a rapid platelet response.

## Introduction

Immune thrombocytopenia (ITP) is an acquired autoimmune disease characterized by low platelet count (<100 × 10^9^/L) and elevated risk of bleeding.^1^ Corticosteroids and intravenous immunoglobulin, the first-line therapies for ITP,^2^ increase platelet count rapidly, but do not offer durable remission in many patients.^3^^,^^4^ Second-line therapies include thrombopoietin receptor agonists (TPO-RAs, e.g., romiplostim, eltrombopag, avatrombopag, and hetrombopag, and recombinant human thrombopoietin [rhTPO]).^5^, ^6^, ^7^, ^8^, ^9^, ^10^ Alternative treatments include splenectomy and agents that inhibit platelet destruction (e.g., anti-CD20 antibody rituximab and spleen tyrosine kinase [Syk] inhibitor fostamatinib).^11^

rhTPO is a full-length, glycosylated TPO.^12^, ^13^, ^14^ It was approved in China in 2010 as a second-line treatment in adult ITP patients at a fixed dose of 300 U/kg/day for up to 14 days.^15^ In a multicenter double-blinded trial,^16^ 14-day treatment with rhTPO at 300 U/kg/day demonstrated superior efficacy *versus* eltrombopag at 25 mg/day in previously treated adults with ITP.^16^ A retrospective study reported the efficacy and safety of rhTPO at a higher dose of 30,000 U/day for 14 days in ITP patients, rendering possible dose titration of rhTPO based on platelet count during treatment.^17^

We conducted a multicenter trial to compare rhTPO versus eltrombopag using a regimen of variable initial doses (300 and 600 U/kg/day for rhTPO *versus* 25 and 50 mg/day for eltrombopag in patients with baseline platelet count of 20–30 × 10^9^/L *versus* <20 × 10^9^/L).

## Methods

### Study design and patients

**Fig. 1:**
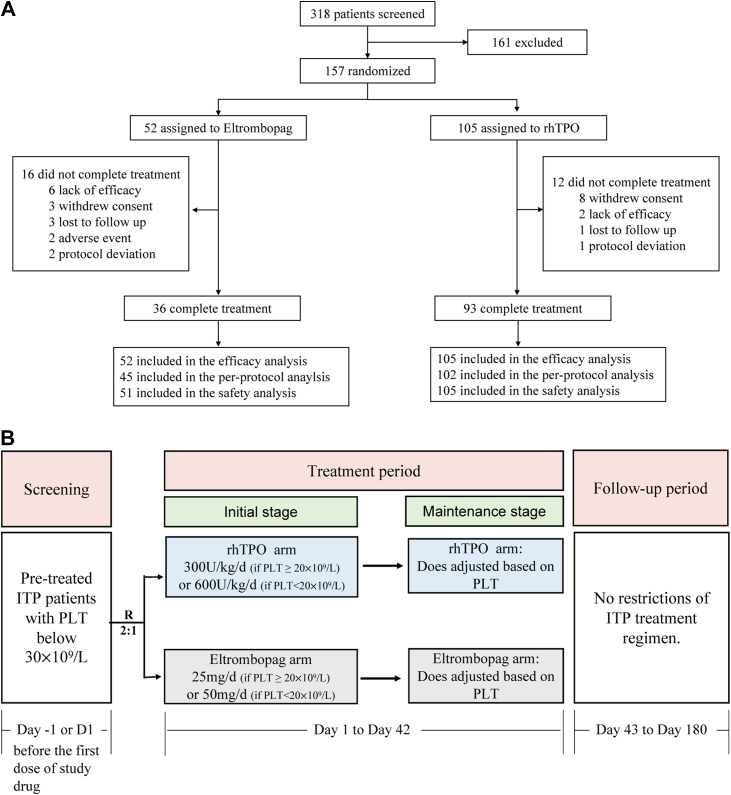
(A) Patient flow through the trial. (B) Trial procedure.

TE-ITP was a randomised, open-label, active comparator-controlled trial in patients with ITP conducted at 12 centers in China. The trial consisted of a 6-week treatment period and a 20-week follow-up period (Fig. 1B), and was conducted in accordance with the Declaration of Helsinki and the International Conference on Harmonisation guidelines for Good Clinical Practice. The trial protocol was approved by all participating centers and registered at ClinicalTrials.gov (NCT05583838, registered on October 12, 2022). All patients provided written informed consent.

Adult patients (≥18 years of age) with previously treated ITP for at least three months were eligible. The platelet count had to be < 30 × 10^9^/L for enrolment. Concomitant medications for ITP, including glucocorticosteroids or immunosuppressants, were allowed, provided that the doses were stable for one month or longer prior to randomisation. Exclusion criteria were: 1) refractory ITP, as defined by failure to respond to first-line therapy, platelet stimulating drugs and rituximab in second-line therapy, splenectomy or postoperative recurrence; 2) having received any platelet increasing drug such as rhTPO, thrombopoietin receptor agonist (TPO-RA), etc. within 30 days; 3) known inadequate response to rhTPO and TPO-RAs; 4) a history of malignancy; 5) serum creatine or total bilirubin >1.5 × upper limit of normal (ULN), alanine aminotransferase and aspartate aminotransferase >3.0 × ULN within two weeks; 6) positive hepatitis C virus antibody or human immunodeficiency virus antibody, positive HBsAg and serum hepatitis B virus DNA >1000 cps/ml; 7) arterial and venous thrombosis or a tendency to thrombosis within one year; 8) pregnant or lactating women.

### Randomisation and treatments

Eligible patients were randomly assigned (2:1) to receive rhTPO or eltrombopag using permuted blocks of 6, using PLAN procedure in SAS (version 9.4), with randomisation stratified according to the baseline platelet count (≥*versus* < 20 × 10^9^/L). The initial dose was 300 or 600 U/kg/day for rhTPO and 25 or 50 mg/day for eltrombopag in patients with platelet count ≥ and <20 × 10^9^/L, respectively. Dose was adjusted weekly to maintain platelet count at 50–250 × 10^9^/L, with a maximum dose of 600 U/kg/day for rhTPO and 75 mg/day for eltrombopag (Table S1).

Rescue treatments, including glucocorticoids, intravenous immunoglobulin and platelet transfusion, were allowed during the treatment period.

### Assessments

Platelet counts were assessed three times weekly until platelet count reached ≥50 × 10^9^/L and weekly thereafter during the treatment period. The patients were followed up until day 180.

Overall response was defined as a platelet count ≥30 × 10^9^/L and at least doubling of the baseline count on two consecutive assessments (separated by ≥ 7 days) with no bleeding. Complete response was defined as a platelet count ≥100 × 10^9^/L on two consecutive assessments (separated by ≥ 7 days) and no bleeding. Time to response (both overall and complete) was calculated from the start of treatment.

Treatment failure was defined as a platelet count <30 × 10^9^/L after four weeks of treatment at the highest dose, a major bleeding event, or a change in therapy due to intolerable toxicities or bleeding (including minor bleeding). Patients who had received rescue treatments were considered as non-responders for the duration of rescue treatment and until platelet count decreased to <50 × 10^9^/L after cessation of rescue treatment.^18^

Bleeding was assessed using the World Health Organization (WHO) bleeding scale (grade 0, no bleeding; grade 1, petechiae; grade 2, mild blood loss; grade 3, gross blood loss; grade 4, debilitating blood loss). Adverse events (AEs) were graded according to the Common Terminology Criteria for Adverse Events (version 5.0).

### Efficacy endpoints

The primary endpoint was the time to first platelet count ≥50 × 10^9^/L, defined as the time from treatment initiation to the first platelet count ≥50 × 10^9^/L at two consecutive visits, separated by at least a two-day interval, with no rescue therapy during the first six weeks of treatment.

Secondary efficacy endpoints included: 1) the proportion of patients with platelet count ≥50 × 10^9^/L at least once by week 6; 2) the proportion of patients who had an overall response or complete response at 1, 4, and 6 weeks; 3) time to response and complete response; 4) the proportion of patients who responded at four or more of the last six visits at four or six months; 5) duration of platelet count ≥50 × 10^9^/L; 6) the median time to treatment failure; 7) the proportion of patients with bleeding at week 1–6; 8) the proportion of patients with reduced or discontinued baseline concomitant treatment for ITP; 9) the proportion of patients requiring rescue therapy.

### Statistical analysis

Sample size was estimated based on the following assumptions: 1) the median time to the first platelet count ≥50 × 10^9^/L at 8 days for rhTPO and 13 days for eltrombopag; 2) 80% power to detect a hazard ratio (HR) of 0.61 using a log-rank test at a two-sided significance level of 0.05. A sample size of 175 patients (rhTPO 117 patients; eltrombopag 58 patients) was anticipated. A pre-planned interim analysis for re-estimating sample size was conducted when the primary efficacy endpoint was available in 105 subjects. The overall type I error (0.05) was controlled using the Peto method (1st alpha 0.001, 2nd alpha 0.050).

Efficacy measures were analyzed based on the intent-to-treat (ITT) population that included all randomised patients. The primary endpoint was also analyzed in the per-protocol population that excluded patients with major protocol deviation as a sensitivity analysis. Safety measures were analyzed in all patients who received at least one dose of the study drug.

The primary endpoint was compared between the two groups using the log-rank test stratified by baseline platelet count (≥*versus* < 20 × 10^9^/L) and displayed using Kaplan Meier curves. For analysis purposes, subjects without a platelet count of ≥50 × 10^9^/L were deemed censored at the end of the treatment period or at the date of withdrawal. Pre-planned subgroup analyses were conducted using a stratified Cox regression model. Key secondary endpoints of platelet response, the proportion of patients who received rescue treatment and who had reduced or interrupted concomitant ITP treatment at baseline, were analyzed using the stratified Cochran-Mantel-Haenszel (CMH) test. Time-to-event secondary endpoints were analyzed similar to the primary endpoint. The duration of platelet count ≥50 × 10^9^/L within six weeks of treatment was analyzed using the Wilcoxon rank sum test. The proportion of patients with bleeding (WHO grade 1–4) at week 1–6 was compared between the two groups using a repeated measures model for binary data with generalized estimating equations method adjusted for the randomisation stratification variable.

A *p*-value of <0.05 was considered statistically significant. All statistical analyses were performed using SAS version 9.4.

### Role of the funding source

The funder of the study had no role in study design, data collection, data analysis, data interpretation, or writing of the report.

## Results

### Patient characteristics

A total of 318 patients were screened between November 22, 2022 and January 16, 2024. The pre-planned interim analysis was conducted in December 2023 when the primary endpoint was available in the first enrolled 105 patients. The analysis revealed that the median time to first platelet count ≥50 × 10^9^/L was 7 days (95% CI 5.0–7.0) in the rhTPO group *versus* 15 days (95% CI 9.0–21.0) in the eltrombopag group (*p* < 0.001). At this point, based on the result of interim analysis, the number of enrolled patients (157) provided 90% power to reject the null hypothesis with an alpha level of 0.05. Accordingly, we stopped further patient recruitment.

A total of 157 patients were randomised (105 and 52 patients in the rhTPO and eltrombopag groups, respectively) (Fig. 1A). The median age of the patients was 52 years (IQR: 36–59), and 66.2% (104/157) were women. The demographic and baseline characteristics of the enrolled patients are shown in Table 1. There were no significant differences in demographic and baseline characteristics between the intervention groups (all *p* > 0.05).

**Table 1:** Demographic and baseline characteristics of the patients.

Characteristics	rhTPO	Eltrombopag	Total
	n = 105	n = 52	n = 157
**Age, median [IQR], years**	53.00 [39.00, 60.00]	48.50 [34.25, 56.00]	52.00 [36.00, 59.00]
**Female sex, n (%)**	69 (65.7)	35 (67.3)	104 (66.2)
**BMI, mean (SD), kg/m^2^**^a^	26.53 (14.87)	25.12 (4.29)	26.07 (12.40)
**Baseline platelet counts**			
Median (IQR), × 10^9^/L	16.0 (9.0–23.0)	16.0 (7.75–23.0)	16.0 (9.0–23.0)
<20 × 10^9^/L^b^	60 (57.1)	30 (57.7)	90 (57.3)
≥20 × 10^9^/L^b^	45 (42.9)	22 (42.3)	67 (42.7)
**Duration of ITP, years; Median (IQR)**	1.0 (0.4–4.9)	1.1 (0.4–6.6)	1.1 (0.4–5.1)
**Classification of ITP, n (%)**			
Persistent ITP^c^	50 (47.6)	25 (48.1%)	75 (47.8%)
Chronic ITP^d^	55 (52.4)	27 (51.9%)	82 (52.2%)
**Concomitant medication at baseline, n (%)**			
No	70 (66.7)	36 (69.2)	106 (67.5)
Glucocorticoid	32 (30.5)	15 (28.8)	47 (29.9)
Immunosuppressant	3 (2.9)	1 (1.9)	4 (2.5)
**Prior ITP treatments, n (%)**			
Glucocorticoid	100 (95.2)	51 (98.1)	151 (96.2)
Intravenous Immunoglobulin	25 (23.8)	14 (26.9)	39 (24.8)
Platelet-stimulating drugs			
rhTPO	33 (31.4)	17 (32.7)	50 (31.8)
TPO receptor agonists	28 (26.7)	17 (32.7)	45 (28.7)
rhIL-11	4 (3.8)	1 (1.9)	5 (3.2)
Rituximab	3 (2.9)	2 (3.8)	5 (3.2)
Chinese herbal medicine	26 (24.8)	16 (30.8)	42 (26.8)
Immunosuppressive drugs^e^	14 (13.3)	5 (9.6)	19 (12.1)
Danazol	12 (11.4)	9 (17.3)	21 (13.4)
Vincristine	0 (0.0)	2 (3.8)	2 (1.3)
Splenectomy	0	0	0
Platelet transfusion	32 (30.5)	14 (26.9)	46 (29.3)
**WHO bleeding scale score**^f^**, n (%)**			
0	60 (57.1)	30 (57.7)	90 (57.3)
1	38 (36.2)	17 (32.7)	55 (35.0)
2	7 (6.7)	5 (9.6)	12 (7.6)

a BMI (body-mass index) is the weight in kilograms divided by the square of the height in meters.

b Stratification factor.

c Persistent ITP is defined as ITP duration of 3–12 months.

d Chronic ITP is defined as ITP duration of over 12 months.

e Include cyclosporin A, sirolimus, mycophenolate mofetil, and cyclophosphamide.

f 0, no bleeding; grade 1, petechiae; grade 2, mild blood loss; grade 3, gross blood loss; grade 4, debilitating blood loss.

The percentage of patients with platelet count <20 × 10^9^/L was 57.1% (60/105) in the rhTPO group *versus* 57.7% (30/52) in the eltrombopag group. 96.2% (151/157) of patients had previously received standard first-line treatment. Corticosteroids were the most frequently reported prior therapy [95.2% (100/105) and 98.1% (51/52) in the rhTPO and eltrombopag groups, respectively]. The proportion of patients with concomitant ITP therapy was 33.3% (35/105) in the rhTPO group and 30.8% (16/52) in the eltrombopag group.

The percentage of patients who did not complete the 6-week treatment was 11.4% (12/105) in the rhTPO group *versus* 30.8% (16/52) in the eltrombopag group. Among 16 patients in the eltrombopag group who did not complete the treatment, 37.5% (6/16) patients discontinued due to lack of efficacy, 18.8% (3/16) withdrew consent, 18.8% (3/16) were lost to follow-up, 12.5% (2/16) experienced adverse events, and 12.5% (2/16) had protocol deviations. In the rhTPO group, the most common reason for treatment discontinuation was withdrawal of consent (66.7%, 8/12), followed by lack of efficacy (16.7%, 2/12), loss to follow-up (8.3%, 1/12), and protocol deviations (8.3%, 1/12).

### Primary efficacy endpoint

**Fig. 2:**
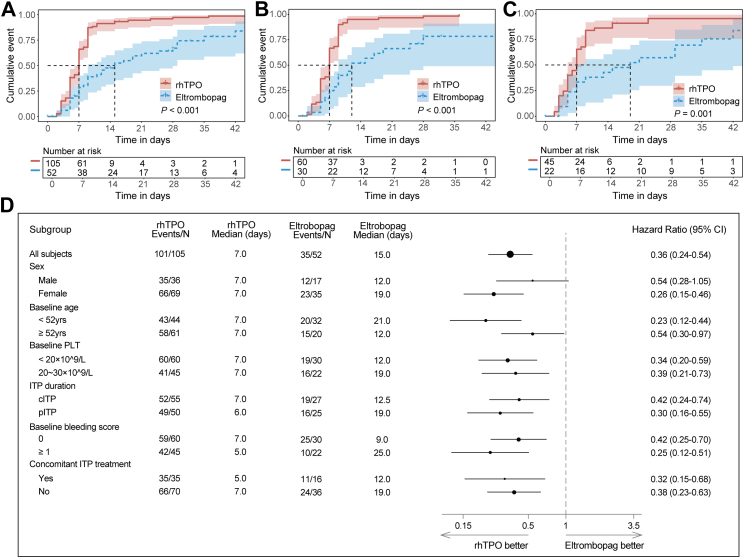
(A) Kaplan–Meier curve of time to the target platelet count (≥50 × 10^9^/L) measured ≥2 days apart in the overall cohort. (B–C) Kaplan–Meier curve of time to the target platelet count according to baseline platelet count <20 × 10^9^/L (B) or ≥ 20 × 10^9^/L (C). (D) Forest plots of treatment responses in prespecified subgroups.

The median time to reach the platelet count ≥50 × 10^9^/L measured at least 2 days apart was 7 days (95% CI 6.0–7.0) in the rhTPO group *versus* 15 days (95% CI 9.0–25.0) in the eltrombopag group (stratified log-rank test *p* < 0.001; Table 2, Fig. 2A). The HR was 0.36 (95% CI, 0.24–0.54, Fig. 2D). In the subgroup analysis that included patients with baseline platelet count <20 × 10^9^/L only, the median time to reach the target platelet count was 7 days (95% CI 5.0–7.0) in the rhTPO group *versus* 12 days (95% CI 9.0–25.0) in the eltrombopag group (log-rank test *p* < 0.001; Fig. 2B; HR 0.34, 95% CI 0.20–0.59, Fig. 2D). In the subgroup analysis that included patients with baseline platelet count ≥20 × 10^9^/L only, the median time to reach the target platelet count was 7 days (95% CI 5.0–7.0) in the rhTPO group *versus* 19 days (95% CI 7.0–35.0) in the eltrombopag group (log-rank test *p* < 0.001, Fig. 2C; HR 0.39, 95% CI 0.21–0.73, Fig. 2D). Shorter time to reach the target platelet count with rhTPO was evident in all other pre-specified subgroup analyses (Fig. 2D). The results from the per-protocol analysis were consistent with the ITT analysis (Figure S1).

**Table 2:** Primary endpoint and secondary efficacy endpoints within the 6-week treatment period.

	rhTPO	Eltrombopag	*p*
**Primary endpoint**			
Time to first platelet count ≥50 × 10^9^/L			
Median (95% CI)	7.0 (6.0–7.0)	15.0 (9.0–25.0)	<0.001^a^
**Secondary endpoints**			
Proportion of patients with platelet count ≥50 × 10^9^/L at least once by week 6, n (%)	101 (96.2)	34 (65.4)	<0.001^b^
Time to response^c^,			
Median days (95% CI)	7.0 (6.0–7.0)	19.0 (9.0–35.0)	<0.001^a^
Response rate, n (%)			
Week 1	67 (63.8)	14 (26.9)	<0.001^b^
Week 2	85 (81.0)	20 (38.5)	<0.001^b^
Week 4	90 (85.7)	30 (57.7)	<0.001^b^
Week 6	92 (87.6)	32 (61.5)	<0.001^b^
Time to complete response^c^, days	13.0 (9.0–30.0)	NA (28.0∼NA)	<0.001^a^
Complete response rate, n (%)			
Week 1	35 (33.3)	5 (9.6)	0.001^b^
Week 2	55 (52.4)	9 (17.3)	<0.001^b^
Week 4	62 (59.0)	17 (32.7)	0.002^b^
Week 6	71 (67.6)	17 (32.7)	<0.001^b^
Duration of platelet count ≥50 × 10^9^/L,			
Median days (IQR)	22.0 (14.0–35.0)	16.0 (2.0–25.0)	<0.001^d^
Patients who reduced or discontinued baseline concomitant treatment for ITP, n (%)	32 (91.4)	13 (81.3)	0.475^b^
Proportion of patients required rescue therapy, n (%)	16 (15.2)	15 (28.8)	0.044^b^
**Proportion of patients with any bleeding (WHO grade 1–4), n (%)**			
OR (95% CI)	0.523 (0.360–0.758)		<0.001^e^
Week 1	6 (5.7)	10 (19.2)	
Week 2	1 (1.0)	3 (5.8)	
Week 3	1 (1.0)	3 (5.8)	
Week 4	3 (2.9)	4 (7.7)	
Week 5	1 (1.0)	1 (1.9)	
Week 6	2 (1.9)	2 (3.8)	

a Stratified log-rank test.

b Stratified CMH.

c Time to response and complete response are defined as the time from the start of study medications to the first occurrence of two consecutive platelet counts ≥30 × 10^9^/L and ≥100 × 10^9^/L, respectively.

d Wilcoxon test.

e Generalized estimating equations method.

### Platelet count during and after the treatment

**Fig. 3:**
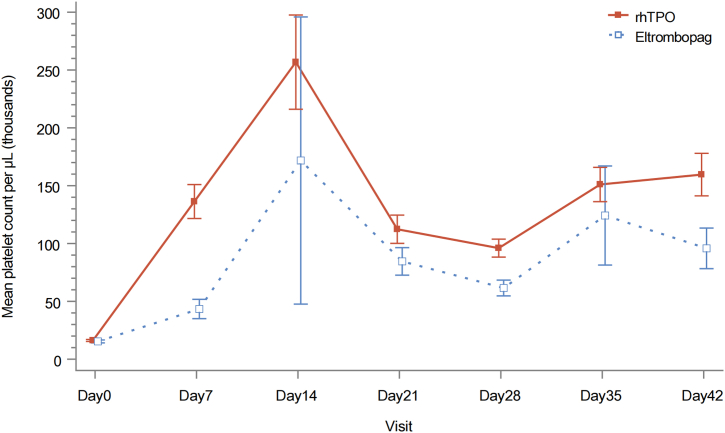
Mean platelet count at each visit. The data are shown as means (standard errors).

The mean platelet count at each study visit is shown in Fig. 3. The percentage of patients achieving platelet count ≥50 × 10^9^/L at least once by week 6 was 96.2% (101/105) and 65.4% (34/52) in the rhTPO and eltrombopag groups, respectively (stratified CMH test, *p* < 0.001) (Table 2). The rhTPO group had higher rate of overall response (63.8% *versus* 26.9%, 81.0% *versus* 38.5%, 85.7% *versus* 57.7% and 87.6% *versus* 61.5% at weeks 1, 2, 4 and 6, respectively), as well as complete response (33.3% *versus* 9.6%, 52.4% *versus* 17.3%, 59.0% *versus* 32.7%, and 67.6% *versus* 32.7% at weeks 1, 2, 4 and 6, respectively) (Table 2). There was no difference in the efficacy assessed at four or six months between two groups (Table S3).

**Fig. 4:**
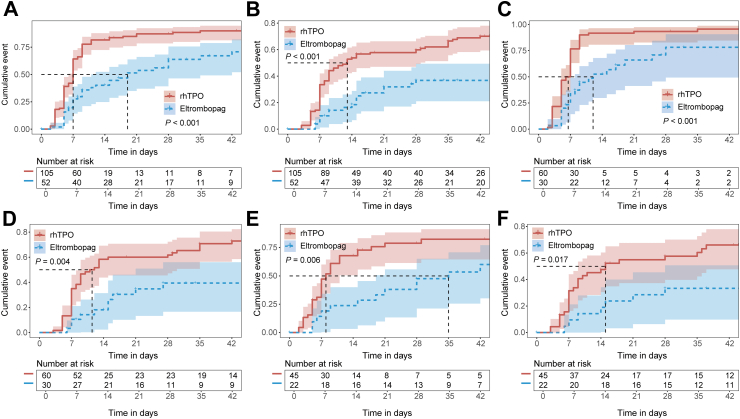
Kaplan–Meier curve of time to response and complete response. (A and B) Kaplan–Meier curve of time to response (A) and complete response (B) in the overall cohort. (C and D) Kaplan–Meier curve of time to response (C) and complete response (D) in patients with baseline platelet count <20 × 10^9^/L. (E and F) Kaplan–Meier curve of time to response (E) and complete response (F) in patients with baseline platelet count≥ 20 × 10^9^/L.

The rhTPO group had shorter time to response (median 7.0 days *versus* 19.0 days; stratified log-rank test *p* < 0.001) as well as time to complete response (13.0 days *versus* not reached; stratified log-rank test *p* < 0.001) in comparison with the eltrombopag group (Fig. 4A and B). The duration of the platelet count staying at ≥ 50 × 10^9^/L was 22.0 days in the rhTPO group *versus* 16.0 days in the eltrombopag group (Wilcoxon rank sum test *p* < 0.001) (Table 2). Subgroup analyses favored rhTPO over eltrombopag in patients regardless of baseline platelet count (Table S2, Fig. 4C–F).

### Bleeding

**Fig. 5:**
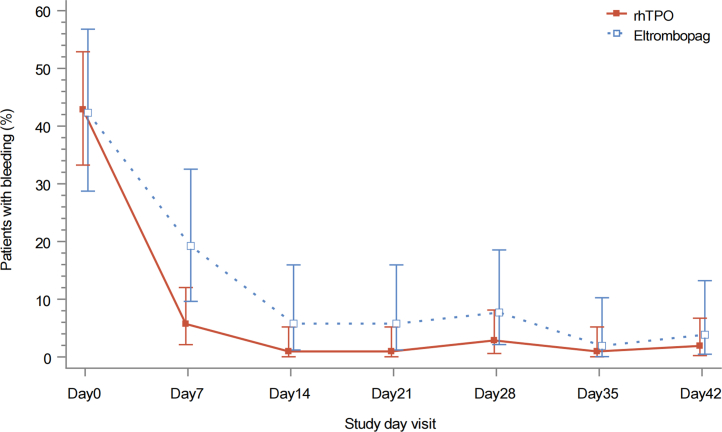
Proportion of patients with bleeding over time. The 95% confidence intervals for each proportion were calculated using the Clopper–Pearson (exact binomial) method.

At baseline, the bleeding rate was comparable between the rhTPO and eltrombopag groups [42.9% (45/105) *versus* 42.3% (22/52)]. There was a decreasing trend in the proportion of patients with bleeding from baseline to week 6 in both groups. In comparison to the eltrombopag group, the rhTPO group had a 47.7% reduction in the risk of bleeding throughout the 6-week treatment period (OR 0.523, 95% CI 0.360–0.758; *p* < 0.001; Table 2, Fig. 5). The temporal profile of the bleeding events also differed between the two groups: in comparison to the eltrombopag group, the remission of bleeding in the rhTPO group tended to occur earlier.

### Treatment failure and rescue therapy

The rate of treatment failure was 6.7% (7/105) in the rhTPO group *versus* 28.8% (15/52) in the eltrombopag group (stratified log-rank test *p* < 0.001). The median time to treatment failure was not reached in either group (Figure S2).

The proportion of patients who discontinued or reduced baseline concomitant ITP treatments was 91.4% (32/35) in the rhTPO group *versus* 81.3% (13/16) in eltrombopag group. The rate of rescue therapy was 15.2% (16/105) in the rhTPO group *versus* 28.8% (15/52) in the eltrombopag group (stratified CMH *p* = 0.044, Table 2, Table S4).

### Safety

During the 6-week treatment period, AEs of any grade occurred in 48 patients (45.7%) in the rhTPO group and 31 (60.8%) in the eltrombopag group. The majority of AEs were grade 1 or 2. No grade 3 or higher AEs occurred in the rhTPO group and one patient (2.0%) in the eltrombopag group had experienced grade 3 AEs (intracranial hemorrhage and cerebral infarction). The most frequent AEs in the rhTPO group were upper respiratory tract infections (10.5%), alanine aminotransferase increased (6.7%), ecchymosis/petechia (5.7%) and blood pressure increased (5.7%). The most frequent AEs in the eltrombopag group were alanine aminotransferase increased (17.6%), upper respiratory tract infections (15.7%), and total bilirubin increased (13.7%) (Table 3). One patient in each group experienced thromboembolic events during the treatment period. rhTPO treatment was suspended in one patient due to deep vein thrombosis, but resumed after antithrombotic therapy. In the eltrombopag group, one patient developed cerebral infarction, leading to discontinuation of eltrombopag treatment and withdrawal from the trial. The patient improved after symptomatic treatment, was discharged from the hospital, and subsequently underwent oral prednisone therapy. No death was reported in either group. Safety profile during the treatment and follow-up period is listed in Table S5.

**Table 3:** Treatment-emergent adverse events within the treatment period.

AE, n (%)	rhTPO (n = 105)					Eltrombopag (n = 51)				
	Any	Grade 1	Grade 2	Grade 3	Grade 4 or 5	Any	Grade 1	Grade 2	Grade 3	Grade 4 or 5
**Any TEAE**	48 (45.7)	33 (31.4)	15 (14.3)	0	0	31 (60.8)	17 (33.3)	13 (25.5)	1 (2.0)	0
**Abnormal liver function**										
ALT increase	7 (6.7)	7 (6.7)	0	0	0	9 (17.6)	8 (15.7)	1 (2.0)	0	0
AST increase	5 (4.8)	5 (4.8)	0	0	0	5 (9.8)	4 (7.8)	1 (2.0)	0	0
ALP increase	3 (2.9)	3 (2.9)	0	0	0	1 (2.0)	1 (2.0)	0	0	0
Blood bilirubin increase	5 (4.8)	5 (4.8)	0	0	0	7 (13.7)	7 (13.7)	0	0	0
**Infection**										
Upper respiratory tract infections	11 (10.5)	5 (4.8)	6 (5.7)	0	0	8 (15.7)	4 (7.8)	4 (7.8)	0	0
Lung infection	1 (1.0)	0	1 (1.0)	0	0	0	0	0	0	0
Ear Infection	1 (1.0)	0	1 (1.0)	0	0	0	0	0	0	0
Tooth infection	0	0	0	0	0	1 (2.0)	0	1 (2.0)	0	0
**Gastrointestinal disorders**										
Anorexia	1 (1.0)	1 (1.0)	0	0	0	0	0	0	0	0
Vomiting	3 (2.9)	2 (1.9)	1 (1.0)	0	0	0	0	0	0	0
Nausea	1 (1.0)	0	1 (1.0)	0	0	1 (2.0)	1 (2.0)	0	0	0
Diarrhea	3 (2.9)	2 (1.9)	1 (1.0)	0	0	2 (3.9)	0	2 (3.9)	0	0
Abdominal discomfort	2 (1.9)	2 (1.9)	0	0	0	2 (3.9)	2 (3.9)	0	0	0
**Pain**										
Injection site pain	1 (1.0)	1 (1.0)	0	0	0	0	0	0	0	0
Myalgia	1 (1.0)	1 (1.0)	0	0	0	2 (3.9)	2 (3.9)	0	0	0
**Bleeding manifestations**										
Petechiae	6 (5.7)	6 (5.7)	0	0	0	2 (3.9)	2 (3.9)	0	0	0
Epistaxis	2 (1.9)	1 (1.0)	1 (1.0)	0	0	3 (5.9)	3 (5.9)	0	0	0
Oral hemorrhage	3 (2.9)	2 (2.9)	0	0	0	2 (3.9)	2 (3.9)	0	0	0
Respiratory tract bleeding	0	0	0	0	0	1 (2.0)^b^	0	1 (2.0)	0	0
Upper gastrointestinal hemorrhage	1 (1.0)^a^	0	1 (1.0)	0	0	0	0	0	0	0
Vagina bleeding	0	0	0	0	0	1 (2.0)	0	1 (2.0)	0	0
Intracranial hemorrhage	0	0	0	0	0	1 (2.0)	0	0	1 (2.0)	0
**Thrombosis**	1 (1.0)	0	1 (1.0)	0	0	1 (2.0)	0	0	1 (2.0)	0
**Anemia**	5 (4.8)	4 (3.8)	1 (1.0)	0	0	5 (9.8)	3 (5.9)	2 (3.9)	0	0
**Fever**	3 (2.9)	3 (2.9)	0	0	0	0	0	0	0	0
**Dizziness**	5 (4.8)	4 (3.8)	1 (1.0)	0	0	2 (3.9)	2 (3.9)	0	0	0
**Headache**	4 (3.8)	4 (3.8)	0	0	0	2 (3.9)	2 (3.9)	0	0	0
**Fatigue**	3 (2.9)	3 (2.9)	0	0	0	3 (5.9)	3 (5.9)	0	0	0
**Insomnia**	0	0	0	0	0	1 (2.0)	1 (2.0)	0	0	0
**Rash**	1 (1.0)	1 (1.0)	0	0	0	1 (2.0)	0	1 (2.0)	0	0
**Edema face**	0	0	0	0	0	2 (3.9)	1 (2.0)	1 (2.0)	0	0
**Blood pressure increase**	6 (5.7)	3 (2.9)	3 (2.9)	0	0	5 (9.8)	2 (3.9)	3 (5.9)	0	0

a This bleeding event occurred on Day 3, before the onset of rhTPO, and was unrelated to rhTPO.

b This bleeding event occurred on Day 3, before the onset of eltrombopag, and was unrelated to eltrombopag.

## Discussions

rhTPO has been shown to be effective in rapidly increasing platelet count in patients with ITP.^12^^,^^19^ However, previous trials mainly focused on the safety and efficacy of rhTPO with a fixed dose of 300 U/kg daily for ITP in a 2-week period, and there was no evidence on the safety and efficacy of consecutive administrations of rhTPO exceeding two weeks using a platelet count-optimised treatment strategy. The current study is the first randomised, controlled trial to compare an optimised administration of rhTPO *versus* eltrombopag for six weeks. The trial results confirmed the advantages of the optimised rhTPO regimen, including rapid elevation and effective maintenance of platelet count at a safe level, a high response rate, a reduced risk of bleeding, and a good safety profile. It is also the first randomised trial to confirm the efficacy of initiating rhTPO at a dose of 600 U/kg.

The median time to the first of two consecutive platelet counts ≥50 × 10^9^/L in the eltrombopag group in this trial (15 days) is comparable to that reported for hetrombopag 5 mg daily in Chinese patients who received previous treatment for ITP.^7^ In our trial, the rhTPO group achieved the target platelet count much faster than the eltrombopag group (7 days *versus* 15 days) regardless of baseline platelet count. This time (7 days) also appears shorter than the 9 days reported for initiating rhTPO at 300 U/kg.^16^ It is also worth noting that the proportion of patients who did not complete the 6-week treatment was higher in the eltrombopag group (30.8%, 16/52) than in the rhTPO group (11.4%, 12/105). In the eltrombopag group, nearly 40% of the discontinuations were due to insufficient efficacy. These findings are consistent with individualized approach in ITP treatment as recommended for TPO-RAs (e.g., romiplostim and eltrombopag).^18^^,^^20^^,^^21^ The findings also encourage using rhTPO tailored to individual patient conditions, particularly in patients with high risk of bleeding and perhaps in the emergency setting.

The more rapid onset of action with rhTPO may be related to the difference in the mechanisms of action between rhTPO and TPO-RAs. rhTPO could act on all stages of megakaryocyte formation, including polyploidy and maturation for rapid platelet release (the time required for megakaryocytes to complete polyploidization, mature and release platelets is approximately 5 days in humans) whereas eltrombopag acts in earlier stages of megakaryocyte formation, i.e., stimulation of progenitor cells and differentiation.^22^^,^^23^

A previous trial demonstrated a higher response rate in adult ITP patients treated with rhTPO at an initial dose of 300 U/kg daily *v* eltrombopag at an initial dose of 25 mg daily for 14 days.^16^ However, it remains an issue of debate whether the initial dose of 25 mg daily for eltrombopag is adequate.^24^ Indeed, several previous studies indicated that higher doses of eltrombopag (either 50 or 75 mg daily) are needed to achieve the treatment goal in some Asian patients.^25^ In this trial, the initial dose of eltrombopag was 25 mg daily and 50 mg daily in patients with platelet count of 20–30 × 10^9^/L and <20 × 10^9^/L, respectively. In addition, the doses of both rhTPO and eltrombopag were adjusted during the treatment period, thus providing further support to the superiority of rhTPO.

The rate of AEs was lower in the rhTPO group *versus* the eltrombopag group during the 6-week treatment period (45.7% *versus* 60.8%). No new safety signal occurred. Notably, the rate of abnormal liver function was lower in the rhTPO group, encouraging the use of rhTPO in ITP patients with hepatic disease. Previous studies showed that although mostly mild, increases in serum aminotransferases and bilirubin have been repeatedly observed in adults using eltrombopag.^26^ This may be due to the inhibition of the organic anion transporter OATP1B1 and a glucuronosyltransferase, UGT1A1, induced by eltrombopag, which may lead to indirect hyperbilirubinemia.^26^^,^^27^ The rate of thromboembolic events was low in both groups (rhTPO 1.0% *versus* eltrombopag 2.0%).

Strengths of this trial were parallel-group design, randomisation stratified by baseline platelet count, and assessment of efficacy and safety for up to six months. This trial still has several limitations. First, it should be noted that the open-label design of this trial may introduce a potential risk of bias. However, we mitigated this risk through centralized randomisation to reduce allocation bias and by applying uniform, predefined response criteria and standardized operating procedures at every site to minimize assessment bias. Second, we did not evaluate changes in the quality of life, which is an important concern among patients with ITP.^28^ Third, patients with refractory ITP were excluded. More clinical trials are required to investigate strategies for refractory ITP. Finally, although we extended the duration of treatment to 6 weeks in this trial, it is still necessary to conduct longer-term clinical trials, such as those lasting 24 weeks, to further verify the sustained efficacy and long-term safety of rhTPO and eltrombopag.

In summary, this trial demonstrated a shorter time to reach the target platelet count ≥50 × 10^9^/L in adult ITP patients undergoing treatment with rhTPO *versus* eltrombopag using a dose and frequency-adjusted protocol. The findings could provide evidence for future treatment guideline recommendations and guide physicians in making decisions on tailored therapy of ITP.

## Contributors

Lei Zhang, Yunfei Chen and Ting Sun designed the research; Yunfei Chen, Ting Sun, Da Gao, Wei Wang and Zeping Zhou analyzed and interpreted the data; Yunfei Chen and Ting Sun wrote the manuscript. Yunfei Chen, Ting Sun, Da Gao, Wei Wang, Zeping Zhou, Guangxun Gao and Lei Zhang accessed and verified the data. All authors contributed to the patient treatment and data collection. All authors critically reviewed and approved the final manuscript.

## Data sharing statement

The datasets used and/or analyzed during the current study are available from the corresponding author upon request.

## Declaration of interests

The authors declared no potential conflicts of interest.

## Appendix A Supplementary data

Supplementary File

Protocol

Statistical Analysis Plan
