# Supplementary material for: Dose-optimised recombinant human thrombopoietin versus eltrombopag in patients with immune thrombocytopenia: a multicenter, randomised controlled trial (The TE-ITP Study)
Source: eClinicalMedicine. 2025 Aug 21;87:103459. doi: 10.1016/j.eclinm.2025.103459 (PMC12396493; doi:10.1016/j.eclinm.2025.103459)
Supplement: Supplementary File [file mmc1.docx]

Supplementary Appendix

Supplement to: Yunfei Chen, Ting Sun, Da Gao, et al. Dose-optimised recombinant human thrombopoietin versus eltrombopag in patients with immune thrombocytopenia: a multicenter, randomised controlled trial (The TE-ITP Study)

**Content**

**1.** **List of investigators** 3

**2.** **Supplementary Methods** 4

**2.1** **Inclusion and Exclusion Criteria** 4

**2.1.1 Inclusion Criteria** 4

**2.1.2 Exclusion Criteria** 4

**2.2** **Randomization and treatments** 5

**2.3** **Definition of response** 5

**2.4** **Secondary efficacy endpoints** 5

**2.5** **Sample size calculation** 6

**2.6** **Interim analysis** 6

**2.7** **Statistical analysis** 7

**2.7.1 Statistical description** 7

**2.7.2 Primary endpoint analysis** 7

**2.7.3 Secondary endpoint analysis** 7

**2.7.4 Safety analysis** 7

**3.** **Supplementary Figures** 9

**4.** **Supplementary Tables** 11

1. **List of investigators**

| **No.** | **Center** | **Principal Investigators** | **Number of Patients** |
| --- | --- | --- | --- |
| 1 | Institution of Hematology and Blood Diseases Hospital, Chinese  Academy of Medical Sciences | Lei Zhang | 75 |
| 2 | The Affiliated Hospital of Inner Mongolia Medical University | Da Gao | 15 |
| 3 | The Second Affiliated Hospital of Harbin Medical University | Wei Wang | 13 |
| 4 | The Second Affiliated Hospital of Kunming Medical University | Zeping Zhou | 10 |
| 5 | The First Affiliated Hospital of Airforce Medical University | Guangxun Gao | 8 |
| 6 | Central Hospital of Xi'an | Yanping Song | 7 |
| 7 | Shaanxi Provincial People's Hospital | Yi Wang | 6 |
| 8 | Henan Cancer Hospital | Hu Zhou | 6 |
| 9 | The Second Affiliated Hospital of Guangxi Medical University | Yinghui Lai | 6 |
| 10 | North China University of Science and Technology Affiliated Hospital | Zhenyu Yan | 6 |
| 11 | The Second Hospital of Dalian Medical University | Jinsong Yan | 4 |
| 12 | The Second Hospital of Tianjin Medical University | Jie Bai | 1 |

1. **Supplementary Methods**
   1. **Inclusion and Exclusion Criteria**

**2.1.1 Inclusion Criteria**

1) Male or female, aged ≥ 18 years;

2) Patients with at least 3 months history of ITP who have received treatment for ITP;

3) Baseline platelet count (PLT) < 30×10^9^/L;

4) Patients receiving concomitant corticosteroids or immunosuppressants for ITP is eligible if the doses are stable for at least 1 month;

5) Signed the informed consent form (ICF).

**2.1.2 Exclusion Criteria**

1) Refractory ITP (refractory ITP is defined as ITP diagnosed on diagnostic reassessment in patients who have failed to response to first-line therapeutic drugs, platelet-stimulating drugs in second-line therapy, and rituximab, or who have failed to response to splenectomy/recurred after surgery);

2) History of arterial or venous thrombus or thrombophilia within the past year;

3) Having received any platelet increasing drug such as rhTPO, thrombopoietin receptor agonist (TPO-RA), etc. within 30 days;

4) Known to have poor efficacy with rhTPO or TPO-RAs;

5) Positive test results for hepatitis C virus antibody and human immunodeficiency virus antibody. Patients who are positive for hepatitis B virus surface antigen and have a quantitative test for hepatitis B virus DNA greater than 1000 cps/ml;

6) Creatinine and total bilirubin levels > 1.5 times the upper limit of normal, alanine aminotransferase and glutamic-oxaloacetic transferase levels > 3.0 times the upper limit of normal within the past 2 weeks;

7) History of malignant tumors;

8) Pregnant or breastfeeding women;

9) Any other conditions deemed inappropriate by the investigator to participate in this study.

- 1. **Randomization and treatments**

Eligible patients were randomly assigned (2:1) to receive rhTPO or eltrombopag using permuted blocks of 6. Randomization was stratified according to the baseline platelet count (≥ *vs.* < 20 × 10^9^/L). The initial dose was 300 and 600 U/kg/day for rhTPO and 25 and 50 mg/day for eltrombopag in patients with platelet count ≥ and < 20 × 10^9^/L, respectively. Dose was adjusted weekly to maintain platelet count at 50-250 × 10^9^/L, with maximum of 600 U/kg/day for rhTPO and 75 mg/day for eltrombopag (Table S1).

Rescue treatments, including glucocorticoids, intravenous globulin and platelet transfusion, were allowed during the treatment period.

- 1. **Definition of response**

1) Overall response was defined as a platelet count ≥ 30 × 10^9^/L and at least doubling of the baseline count on two consecutive assessments (separated by ≥ 7 days) with no bleeding.

2) Complete response was defined as a platelet count ≥ 100× 10^9^/L on two consecutive assessments (separated by ≥ 7 days) and no bleeding.

3) Time to response (both overall and complete) was calculated from the start of treatment.

4) Treatment failure was defined as a platelet count < 30 × 10^9^/L after four weeks of treatment at the highest dose, a major bleeding event, or a change in therapy due to intolerable toxicities or bleeding (including minor bleeding).

- 1. **Secondary efficacy endpoints**

1) The proportion of patients with platelet count ≥ 50 × 10^9^/L at least once by week 6;

2) The proportion of patients who had an overall response or complete response at 1, 4, and 6 weeks;

3) Time to response and complete response;

4) The proportion of patients who responded at four or more of the last six visits at 4 or 6 months;

5) Duration of platelet count ≥ 50 × 10^9^/L;

6) The median time to treatment failure;

7) The proportion of patients with bleeding at week 1 to 6;

8) The proportion of patients with reduced or discontinued baseline concomitant treatment for ITP;

9) The proportion of patients requiring rescue therapy.

- 1. **Sample size calculation**

Sample size was estimated based on the following assumptions: 1) the median time to the first platelet count ≥ 50 × 10^9^/L at 8 days for rhTPO and 13 days for eltrombopag; 2) 80% power to detect a hazard ratio (HR) of 0·61 using a log-rank test at a two-sided significance level of 0·05. A sample size of 175 patients (rhTPO 117 patients; eltrombopag 58 patients) was anticipated.

- 1. **Interim analysis**

A pre-planned interim analysis for re-estimating sample size was conducted when the primary efficacy endpoint was available in 105 subjects. The overall type I error (0·05) was controlled using the Peto method (1st alpha 0·001, 2nd alpha 0·050). Sample size will be re-estimated after the interim analysis.

- 1. **Statistical analysis**

**2.7.1 Statistical description**

The quantitative data is described statistically using the number of cases, mean, standard deviation (SD), median, minimum, and maximum. The statistical description of categorical variables is expressed using various patients’ cases and percentages. Unless otherwise specified, bilateral 95% is used as the width of all confidence intervals and the significance level of statistical tests. A *p*-value of < 0·05 was considered statistically significant. All statistical analyses were performed using SAS version 9.4.

**2.7.2 Primary endpoint analysis**

Efficacy measures were analyzed based on the intent-to-treat (ITT) population that included all randomized patients. The primary endpoint was also analyzed in the per-protocol population that excluded patients with major protocol deviation as a sensitivity analysis. Safety measures were analyzed in all patients who received at least one dose of the study drug.

**2.7.3 Secondary endpoint analysis**

Key secondary endpoints of platelet response, the proportion of patients who received rescue treatment and who had reduced or interrupted concomitant ITP treatment at baseline, were analyzed using the stratified Cochran-Mantel-Haenszel (CMH) test‌. Time-to-event secondary endpoints were analyzed similar to the primary endpoint. The duration of platelet count ≥ 50 × 10^9^/L within six weeks of treatment was analyzed using the Wilcoxon rank sum test. The proportion of patients with bleeding (WHO grade 1-4) at week 1 to 6 was compared between the two groups using a repeated measures model for binary data with generalized estimating equations method adjusted for the randomization stratification variable.

**2.7.4 Safety analysis**

Safety endpoints are analyzed in the SS. Adverse events are graded with CTCAE 5.0. The number of events, number of subjects with events, and incidence are summarized for adverse events, adverse reactions, serious adverse events and serious adverse reactions.

1. **Supplementary Figures**


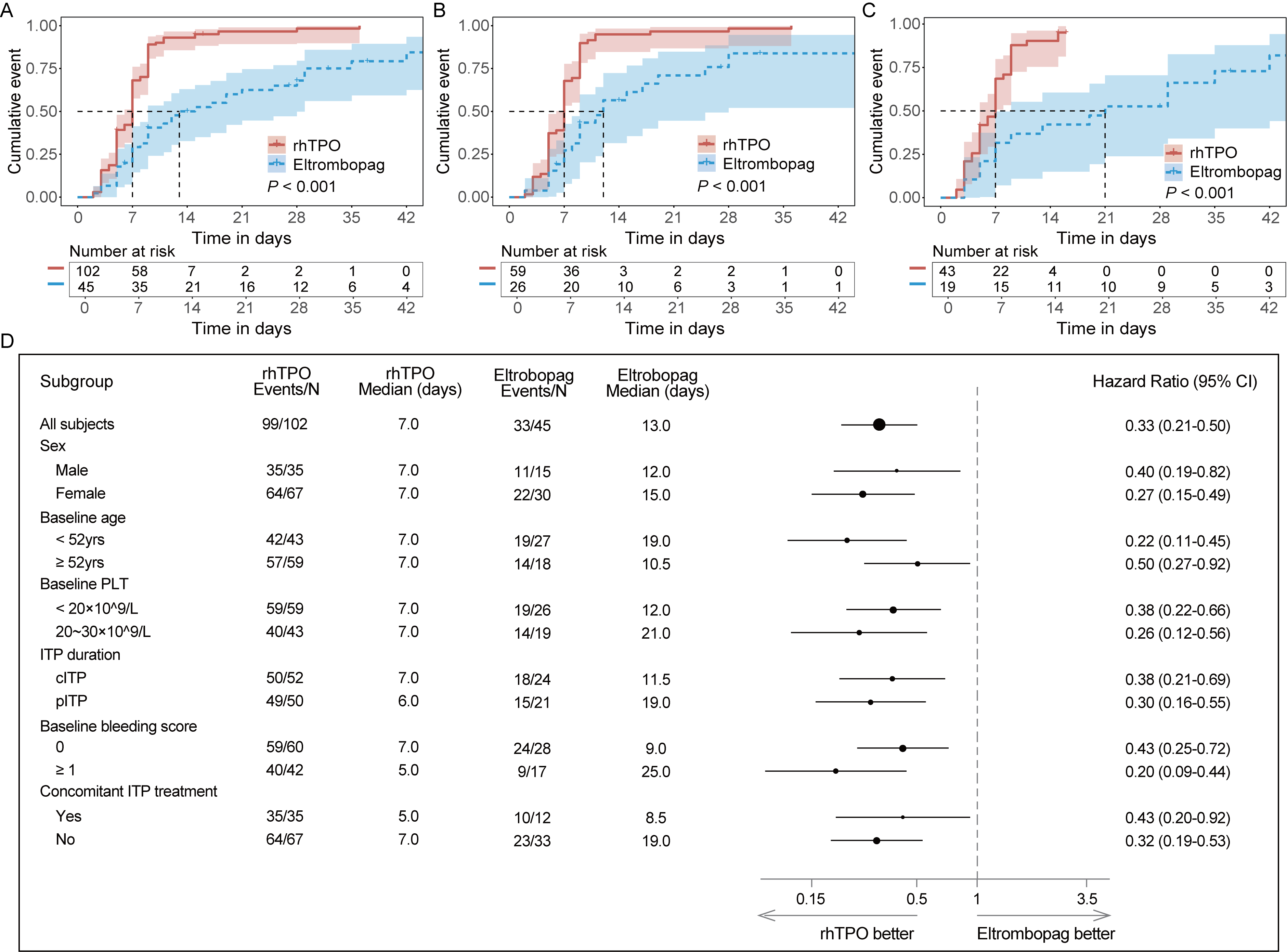


Figure S1: (A) Kaplan-Meier curve of time to the target platelet count (≥ 50 × 10^9^/L) measured ≥ 2 days apart in the overall population of per-protocol analysis. (B-C) Kaplan-Meier curve of time to the target platelet count according to baseline platelet count < 20 × 10^9^/L (B) or ≥ 20 × 10^9^/L (C). (D) Forest plots of treatment responses in prespecified subgroups.


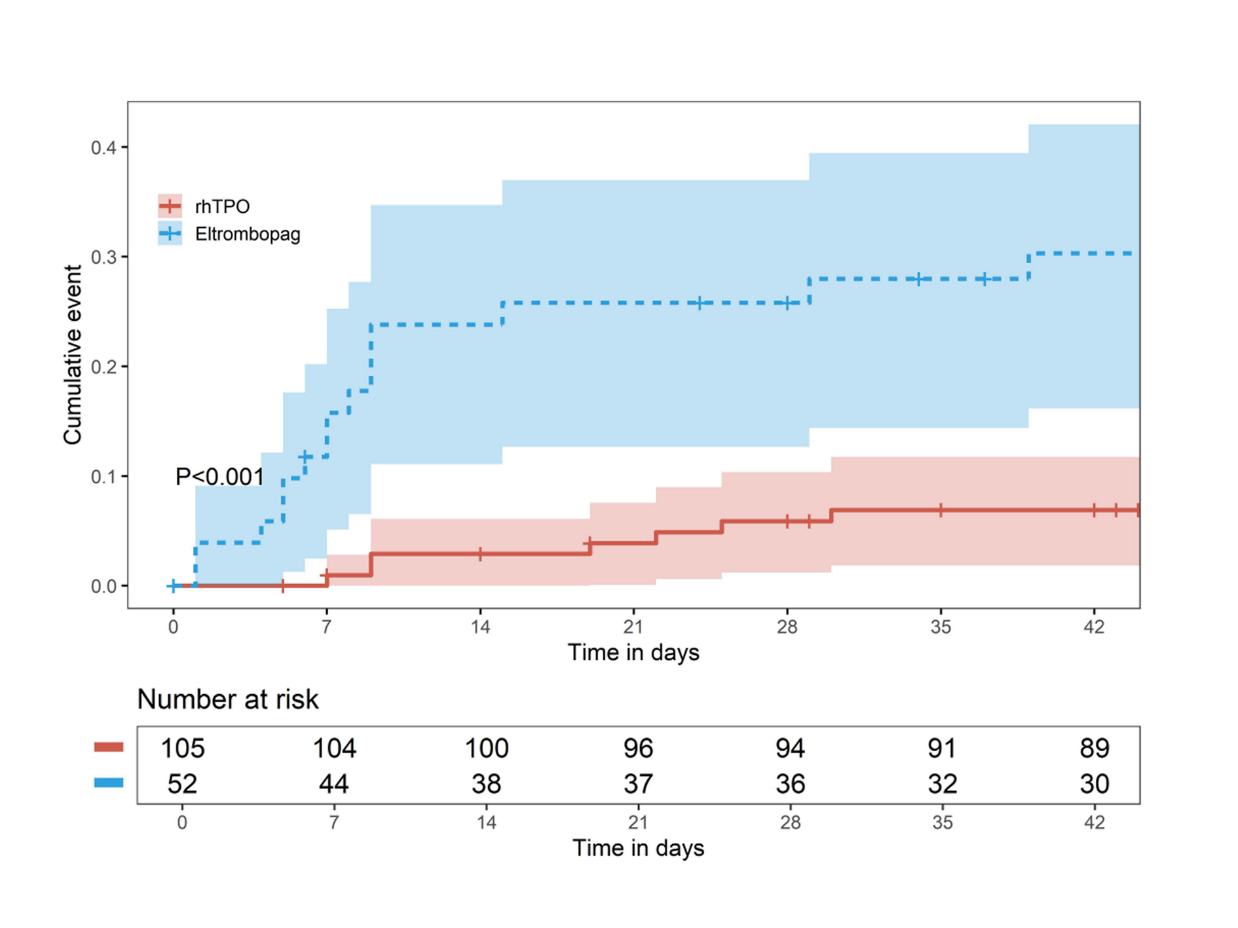


Figure S2: Kaplan-Meier curve of time to treatment failure in the overall cohort.

1. **Supplementary Tables**

| **Table S1. Dose adjustments for rhTPO and eltrombopag during the 6-week treatment period.** | | | | |
| --- | --- | --- | --- | --- |
|  | **Platelet count** | | | |
|  | < 50×10^9^/L | ≥ 50-150×10^9^/L | > 150-250 ×10^9^/L | > 250×10^9^/L |
| **rhTPO** | Increase the daily dose to 300-600 U/kg/day | 600U/kg once every other day | 450 U/kg once every other day | Stop rhTPO until the platelet count is ≤ 100×10^9^/L |
| **Eltrombopag** | Increase the daily dose by 25 mg to a maximum of 75 mg/day. | Maintain the effective dosage | Decrease the daily dose by 25 mg or reduce the frequency | Stop eltrombopag until the platelet count is ≤ 100×10^9^/L |

| **Table S2. Treatment responses stratified by baseline platelet count within the 6-week treatment period.** | | | | | | |
| --- | --- | --- | --- | --- | --- | --- |
|  | **Baseline platelet count** | | | | | |
|  | **<20×10^9^/L** | | | **≥20×10^9^/L** | | |
|  | **rhTPO** | **Eltrombopag** | ***p*** | **rhTPO** | **Eltrombopag** | ***p*** |
| **Time to response^*^, days** |  |  |  |  |  |  |
| Median (95% CI) | 6.5(5.0-7.0) | 12.0(7.0-25.0) | <0.001^†^ | 8.0(7.0-11.0) | 35.0(15.0-NA) | 0.006^†^ |
| **Response rate, n (%)** |  |  |  |  |  |  |
| Week 1 | 46(76.7) | 10(33.3) | <0.001 | 21(46.7) | 4(18.2) | 0.024 |
| Week 2 | 55(91.7) | 15(50.0) | <0.001 | 30(66.7) | 5(22.7) | <0.001 |
| Week 4 | 56(93.3) | 20(66.7) | 0.002 | 34(75.6) | 10(45.5) | 0.015 |
| Week 6 | 57(95.0) | 20(66.7) | <0.001 | 35(77.8) | 12(54.5) | 0.051 |
| **Time to complete response*, days** | 11.5(8.0~29.0) | NA (16.0-NA) | 0.004^†^ | 15.0(9.0-37.0) | NA (21.0-NA) | 0.017^†^ |
| **Complete response rate, n (%)** |  |  |  |  |  |  |
| Week 1 | 21(35.0) | 3(10.0) | 0.011 | 14(31.1) | 2(9.1) | 0.047 |
| Week 2 | 34(56.7) | 5(16.7) | <0.001 | 21(46.7) | 4(18.2) | 0.024 |
| Week 4 | 37(61.7) | 10(33.3) | 0.011 | 25(55.6) | 7(31.8) | 0.068 |
| Week 6 | 43(71.7) | 10(33.3) | <0.001 | 28(62.2) | 7(31.8) | 0.019 |
| **Duration of platelet count ≥ 50×10^9^/L, days** |  |  |  |  |  |  |
| Median (IQR) | 19.0(14.0-32.0) | 16.0(0-21.0) | 0.012 ^¶^ | 27.0(17.0-35.0) | 16.5(7.0-33.0) | 0.053 ^¶^ |
| ^*^Time to response and complete response are defined as the duration between the start of study medications to the first occurrence of two consecutive platelet counts ≥ 30×10^9^/L and ≥100×10^9^/L, respectively. | | | | | | |
| ^†^Log-rank test. | | | | | | |
| ^‡^Chi-square test. | | | | | | |

| **Table S3. The proportion of patients who responded at four or more of the last six visits at 4 or 6 months.** | | |
| --- | --- | --- |
|  | **rhTPO** | **Eltrombopag** |
| 4 months, n (%) | 49(46.7) | 18(34.6) |
| 6 months, n (%) | 51(48.6) | 18(34.6) |

| **Table S4. The type of rescue therapy.** | | |
| --- | --- | --- |
| **Rescue therapy, n (%)** | **rhTPO** | **Eltrombopag** |
|  | **n=16** | **n=15** |
| Glucocorticoid | 0(0) | 6(11.5) |
| Intravenous immunoglobin | 2(1.9) | 3(5.8) |
| Platelet transfusion | 15(14.3) | 14(26.9) |

| **Table S5. Treatment-emergent adverse events within the treatment period and the follow up period.** | | | | | | | | | | |
| --- | --- | --- | --- | --- | --- | --- | --- | --- | --- | --- |
| **AE，n(%)** | **rhTPO(n=105)** | | | | | **Eltrombopag (n=51)** | | | | |
|  | **Any** | **Grade1** | **Grade 2** | **Grade3** | **Grade 4 or 5** | **Any** | **Grade1** | **Grade 2** | **Grade3** | **Grade 4 or 5** |
| **Any TEAE** | 56(53.3) | 35(33.3) | 19(18.1) | 2(1.9) | 0 | 36(70.6) | 18(35.3) | 17(33.3) | 1(2.0) | 0 |
| **Abnormal liver function** |  |  |  |  |  |  |  |  |  |  |
| ALT increase | 13(12.4) | 11(10.5) | 1(1.0) | 1(1.0) | 0 | 11(21.6) | 9(17.6) | 2(3.9) | 0 | 0 |
| AST increase | 9(8.6) | 8(7.6) | 1(1.0) | 0 | 0 | 7(13.7) | 6(11.8) | 1(2.0) | 0 | 0 |
| ALP increase | 3(2.9) | 3(2.9) | 0 | 0 | 0 | 4(7.8) | 4(7.8) | 0 | 0 | 0 |
| Blood bilirubin increase | 5(4.8) | 5(4.8) | 0 | 0 | 0 | 7(13.7) | 7(13.7) | 0 | 0 | 0 |
| **Infection** |  |  |  |  |  |  |  |  |  |  |
| Upper respiratory tract infection | 13(12.4) | 6(5.7) | 7(6.7) | 0 | 0 | 8(15.7) | 4(7.8) | 4(7.8) | 0 | 0 |
| Lung infection | 2(1.9) | 0 | 1(1.0) | 1(1.0) | 0 | 0 | 0 | 0 | 0 | 0 |
| Ear infection | 1(1.0) | 0 | 1(1.0) | 0 | 0 | 0 | 0 | 0 | 0 | 0 |
| Tooth infection | 0 | 0 | 0 | 0 | 0 | 1(2.0) | 0 | 1(2.0) | 0 | 0 |
| Urinary tract infection | 0 | 0 | 0 | 0 | 0 | 1(2.0) | 1(2.0) | 0 | 0 | 0 |
| **Gastrointestinal disorders** |  |  |  |  |  |  |  |  |  |  |
| Anorexia | 2(1.9) | 1(1.0) | 1(1.0) | 0 | 0 | 0 | 0 | 0 | 0 | 0 |
| Vomiting | 3(2.9) | 2(1.9) | 1(1.0) | 0 | 0 | 0 | 0 | 0 | 0 | 0 |
| Nausea | 1(1.0) | 0 | 1(1.0) | 0 | 0 | 1(2.0) | 1(2.0) | 0 | 0 | 0 |
| Diarrhea | 3(2.9) | 2(1.9) | 1(1.0) | 0 | 0 | 2(3.9) | 0 | 2(3.9) | 0 | 0 |
| Abdominal discomfort | 3(2.9) | 3(2.9) | 0 | 0 | 0 | 3(5.9) | 3(5.9) | 0 | 0 | 0 |
| **Pain** |  |  |  |  |  |  |  |  |  |  |
| Injection site pain | 1(1.0) | 1(1.0) | 0 | 0 | 0 | 0 | 0 | 0 | 0 | 0 |
| Myalgia | 1(1.0) | 1(1.0) | 0 | 0 | 0 | 2(3.9) | 2(3.9) | 0 | 0 | 0 |
| **Bleeding manifestations** |  |  |  |  |  |  |  |  |  |  |
| Petechiae | 10(9.5) | 10(9.5) | 0 | 0 | 0 | 2(3.9) | 2(3.9) | 0 | 0 | 0 |
| Epistaxis | 5(4.8) | 4(3.8) | 1(1.0) | 0 | 0 | 3(5.9) | 3(5.9) | 0 | 0 | 0 |
| Oral hemorrhage | 4(3.8) | 4(3.8) | 0 | 0 | 0 | 2(3.9) | 2(3.9) | 0 | 0 | 0 |
| Respiratory tract bleeding | 0 | 0 | 0 | 0 | 0 | 1(2.0) | 0 | 1(2.0) | 0 | 0 |
| Upper gastrointestinal hemorrhage | 1(1.0) | 0 | 1(1.0) | 0 | 0 | 0 | 0 | 0 | 0 | 0 |
| Vagina bleeding | 0 | 0 | 0 | 0 | 0 | 1(2.0) | 0 | 1(2.0) | 0 | 0 |
| Intracranial hemorrhage | 0 | 0 | 0 | 0 | 0 | 1(2.0) | 0 | 0 | 1(2.0) | 0 |
| **Thrombosis** | 1(1.0) | 0 | 1(1.0) | 0 | 0 | 1(2.0) | 0 | 0 | 1(2.0) | 0 |
| **Anemia** | 6(5.7) | 4(3.8) | 2(1.9) | 0 | 0 | 7(9.8) | 3(5.9) | 4(7.8) | 0 | 0 |
| **Fever** | 3(2.9) | 3(2.9) | 0 | 0 | 0 | 0 | 0 | 0 | 0 | 0 |
| **Dizziness** | 5(4.8) | 4(3.8) | 1(1.0) | 0 | 0 | 2(3.9) | 2(3.9) | 0 | 0 | 0 |
| **Headache** | 4(3.8) | 4(3.8) | 0 | 0 | 0 | 2(3.9) | 2(3.9) | 0 | 0 | 0 |
| **Fatigue** | 4(3.8) | 4(3.8) | 0 | 0 | 0 | 3(5.9) | 3(5.9) | 0 | 0 | 0 |
| **Insomnia** | 0 | 0 | 0 | 0 | 0 | 1(2.0) | 1(2.0) | 0 | 0 | 0 |
| **Rash** | 1(1.0) | 1(1.0) | 0 | 0 | 0 | 2(3.9) | 1(2.0) | 1(2.0) | 0 | 0 |
| **Edema face** | 0 | 0 | 0 | 0 | 0 | 2(3.9) | 1(2.0) | 1(2.0) | 0 | 0 |
| **Blood pressure increase** | 7(6.7) | 3(2.9) | 4(3.8) | 0 | 0 | 6(11.8) | 2(3.9) | 4(7.8) | 0 | 0 |
