# Supplementary material for: Dose-optimised recombinant human thrombopoietin versus eltrombopag in patients with immune thrombocytopenia: a multicenter, randomised controlled trial (The TE-ITP Study)
Source: eClinicalMedicine. 2025 Aug 21;87:103459. doi: 10.1016/j.eclinm.2025.103459 (PMC12396493; doi:10.1016/j.eclinm.2025.103459)
Supplement: Protocol [file mmc2.pdf]

Clinical Study Protocol

**Protocol Title:**	A Randomized Controlled Study Comparing the Efficacy and Safety of Optimized rhTPO Regimen versus Eltrombopag in the Pre-treated Adult Patients with ITP (TE-ITP)
**Protocol No.:**	IIT2022037-EC-1
**Principal Investigator:**	Lei Zhang
**Tel**	022-23909083
**Study site:**	Institute of Hematology & Blood Disease Hospital, Chinese Academy of Medical Sciences & Peking Union Medical College
**Address:**	No. 288 Nanjing Road, Heping District, Tianjin
**Version No.:**	1.1
**Version Date:**	2023.9.26

Confidentiality Statement

This document contains confidential information of the Institute of Hematology & Blood Disease Hospital, Chinese Academy of Medical Sciences & Peking Union Medical College, and is intended for the purposes of this clinical study only. Any disclosure is not permitted to anyone other than the study participants and members of the Institutional Review Board. This information will not be used for any purpose other than evaluating or conducting clinical study without written authorization from the Institute of Hematology & Blood Disease Hospital, Chinese Academy of Medical Sciences & Peking Union Medical College.

Statement of Investigator

I am familiar with this study protocol, confirm that it includes necessary contents for study implementation, specifies the responsibilities related to the study protocol. I agree to fulfill my duties in accordance with Chinese laws and regulations, the Declaration of Helsinki, Good Clinical Practice, and this study protocol, and will not carry out the study-specified procedures until the Ethics Committee approves the protocol and the subjects sign the informed consent. If modifications are required to the protocol, the revised protocol must be approved by the Ethics Committee before implementation, unless measures must be taken to protect the safety, rights, and interests of the subjects. I understand and comply with the maintenance requirements of the original.

____ ______________________________

Name of Principal Investigator Date

Study site: Institute of Hematology & Blood Disease Hospital, Chinese Academy of Medical Sciences & Peking Union Medical College

Table of Contents

ABBREVIATIONS 1

1 OVERVIEW 1

2 BACKGROUND 4

2.1 Therapeutic Goals and Principles for Primary Immune Thrombocytopenia (ITP) 4

2.2 Review of Clinical Application of rhTPO in the Treatment of Adult ITP 5

2.3 A Brief Analysis of the Need for Optimized rhTPO Regimen for the Treatment of ITP in Clinical Practice 6

2.4 Review of Head-to-Head Study of rhTPO versus Eltrombopag in the Treatment of Adult ITP 6

3 STUDY OBJECTIVE 7

4 STUDY DESIGN 7

4.1 Overall Design 7

4.2 Rationale of Study Design 7

4.3 Sample Size 12

4.4 Interim Analysis 12

5 STUDY POPULATION 12

5.1 Inclusion Criteria 12

5.2 Exclusion Criteria 13

5.3 Criteria for Dropout and Withdrawal 13

5.4 Elimination Criteria 14

6 INVESTIGATIONAL DRUG 14

7 DOSAGE REGIMEN 14

8 PROHIBITED MEDICATIONS 16

9 CONCOMITANT THERAPY 16

10 SCHEDULE OF VISITS 16

10.1 Screening/Baseline 16

10.2 Treatment Period 17

10.3 Follow-up Period 17

11 EVALUATION ENDPOINTS 19

11.1 Efficacy Endpoints 19

11.2 Safety Endpoints 19

12 ADVERSE EVENTS AND SERIOUS ADVERSE EVENTS 20

12.1 Adverse Event (AE) 20

12.2 Serious Adverse Event 21

12.3 Pre-evaluation of Project Risks and its Mitigation 21

13 DATA MANAGEMENT 22

14 STATISTICAL ANALYSIS 22
14.1 Statistical Analysis Set 22
14.2 Statistical Analysis Method 22
15 ETHICAL REVIEW 23
15.1 Ethical Requirements 23
15.2 Informed Consent 23
15.3 Confidentiality of Subjects 23
16 STUDY MANAGEMENT 24
16.1 Training 24
16.2 Quality Control and Assurance 24
17 CONFIDENTIALITY AND PUBLICATION OF STUDY RESULTS 24
18 REFERENCES 24
19 APPENDICES 27
Appendix 1: World Health Organization (WHO) Bleeding Scoring System 27
Appendix 2 Clinical Laboratory Examination Evaluation List 28

ABBREVIATIONS

Abbreviation/Acronym	Explanation
ADA	Anti-drug antibody
AE	Adverse event
ALT	Alanine aminotransferase
AST	Aspartate aminotransferase
c-MPL	C-Mannosylation of thrombopoietin receptor
Cr	Creatinine
CR	Complete response
CRF	Case report form
CTCAE	Common terminology criteria for adverse events
FAS	Full analysis set
HSC	Hematopoietic stem cell
IIT	Investigator initiated trial
ITP	Immune thrombocytopenia
IVIg	Intravenous immunoglobulin
MPV	Mean platelet volume
NAB	Neutralizing antibody
NR	No response
R	Response
RBC	Red blood cell
rhTPO	Recombinant human thrombopoietin
SAE	Severe adverse event
SS	Safety analysis
TCP	Thrombocytopenia
TPO	Thrombopoietin
TPO-RA	Thrombopoietin receptor agonist
TTF	Time to treatment failure
PLT	Platelet
PP	Per-protocol analysis
WBC	White blood cell
WHO	World Health Organization

OVERVIEW

Investigational Drug Name: Recombinant Human Thrombopoietin (rhTPO) Injection	Clinical Study Stage: Post Marketing IIT Study
Study Title: A Randomized Controlled Study Comparing the Efficacy and Safety of Optimized rhTPO Regimen versus Eltrombopag in the Pre-treated Adult Patients with ITP (TE-ITP)	
Planned Study Duration: October 2022 to September 2024	
Objective:<br>To evaluate the efficacy and safety of optimized rhTPO regimen versus Eltrombopag in the pre-treated adult patients with primary immune thrombocytopenia (ITP) for 6 weeks	
Study population:<br>Pre-treated adult patients with ITP	
Number of subjects planned to be enrolled:<br>175 subjects	
Overview of Study Design:<br>This study is a randomized, open-label, active controlled prospective study that uses a superiority trial design to validate the efficacy and safety of optimized rhTPO regimen vs. the conventional Eltrombopag treatment, and thus evaluate the rationality and clinical benefits of optimized rhTPO regimen.	
Inclusion criteria:<br>Subjects eligible for this study must meet all of the following criteria:<br>1) Male or female, aged ≥ 18 years;<br>2) Patients with at least 3 months history of ITP who have received treatment for ITP;<br>3) Baseline platelet count (PLT) < 30×10^9/L;<br>4) Patients receiving concomitant corticosteroids or immunosuppressants for ITP is eligible if the doses are stable for at least 1 month;<br>5) Signed the informed consent form (ICF)	
Exclusion criteria:<br>Subjects who meet any of the following criteria must be excluded from this study:<br>1) Refractory ITP (refractory ITP is defined as ITP diagnosed on diagnostic reassessment in patients who have failed to response to first-line therapeutic drugs, platelet-stimulating drugs in second-line therapy, and rituximab, or who have failed to response to splenectomy/recurred after surgery);<br>2) History of arterial or venous thrombus or thrombophilia within the past year;<br>3) Having received any platelet increasing drug such as rhTPO, thrombopoietin receptor agonist(TPO-RA), etc. within 30 days;<br>4) Known to have poor efficacy with rhTPO or TPO-RAs;<br>5) Positive test results for hepatitis C virus antibody and human immunodeficiency virus antibody. Patients who are positive for hepatitis B virus surface antigen and have a quantitative test for hepatitis B virus DNA greater than 1000 cps/ml;<br>6) Creatinine and total bilirubin levels > 1.5 times the upper limit of normal, alanine aminotransferase and glutamic-oxaloacetic transferase levels > 3.0 times the upper limit of normal within the past 2 weeks;<br>7) History of malignant tumors;<br>8) Pregnant or breastfeeding women;<br>9) Any other conditions deemed inappropriate by the investigator to participate in this study	
Investigational Drug: Recombinant Human Thrombopoietin (rhTPO) Injection	

Trade Name: TPIAO®<br>Manufacturer: Shenyang Sunshine Pharmaceutical Co., Ltd.<br>Strengths/Approval No.:<br>7500 U/1 mL/vial, GYZZ S20050049<br>15000 U/1 mL/vial, GYZZ S20050048<br>Route of Administration: Subcutaneous administration<br>Storage: 2 to 8 °C, protected from light<br>Study Drug: Eltrombopag Olamine Tablets<br>Trade Name: Revolade®<br>Manufacturer: Novartis Pharma Schweiz AG<br>Strengths/Approval No.:<br>Imported Drug Registration Certificate No.<br>25 mg (calculated as $C_{25}H_{22}N_4O_4$) H20170387<br>50 mg (calculated as $C_{25}H_{22}N_4O_4$) H20170388<br>Route of Administration: oral<br>Storage: Store below 30 °C away from children
Dosage regimen<br>General principles during the 6-week treatment period:<br>● During this period, the dose of study drug will be adjusted weekly based on platelet response in both groups, with a maximum dose of 600 U/kg/day in the rhTPO group and 75 mg/day in the eltrombopag group;<br>● The initial dose is determined by baseline PLT in both groups, as described in the “Initial Dose Regimen” for each group;<br>● After PLT reaches ≥ 50×10^9/L, the dose of the two groups is adjusted according to the platelet level, as described in the “Dose Adjustment Regimen” for each group;<br>● The dose of study drugs may be adjusted by the investigator temporarily or in advance, or alternatively used with adjacent doses when necessary, according to the treatment response and safety of patients;<br>rhTPO group:<br>● Initial dose regimen: rhTPO will be administered at an initial dose of 300 U/kg or 600 U/kg by the investigator based on patients’ PLT and treatment history; for patients with a PLT $\geq 20\times10^9$/L and $< 30\times10^9$/L and no active bleeding, it should be administered at 300U/kg/day continuously; for those with a PLT $< 20\times10^9$/L or active bleeding, it should be administered at 600 U/kg/day continuously; for those with a PLT $< 50\times10^9$/L after 7 days of treatment, it should be administered at 600 U/kg/day continuously; rhTPO is administered subcutaneously;<br>● Dose adjustment regimen: The rhTPO dose is adjusted weekly according to PLT level; if the PLT is $\geq 50\times10^9$/L and $\leq 150\times10^9$/L, rhTPO should be administered at 600 U/kg every other day; if the PLT is $> 150\times10^9$/L and $< 250\times10^9$/L, rhTPO should be administered at 450 U/kg every other day for those who need continued administration; if the PLT is $> 250\times10^9$/L, rhTPO should be discontinued and reinitiated until the PLT is $\leq 100\times10^9$/L.

Eltrombopag group:

- Initial dose regimen: Eltrombopag will be administered at an initial dose of 25 mg or 50 mg/day by the investigator according to baseline PLT and treatment history; for patients with a PLT ≥ 20×10^9/L and ＜ 30×10^9/L and no active bleeding, it should be administered at 25 mg/day continuously; for those with a PLT ＜20×10^9/L or active bleeding, it should be administered at 50 mg/day continuously; for those with a PLT ＜50×10^9/L after 7 days of treatment, the daily dose should be increased by 25 mg to a maximum of 75 mg/day; Eltrombopag is administered orally;
- Dose adjustment regimen: The Eltrombopag dose is adjusted weekly based on PLT level; if the PLT is ≥ 50×10^9/L and ≤ 150×10^9/L, the effective dose should be maintained with a maximum dose of 75 mg/day; if the PLT is > 150×10^9/L and < 250×10^9/L, the dose should be reduced to the adjacent lower dose (such as 50 mg/day reduced to 25 mg/day) or the frequency reduced (such as 25 mg/day reduced to 25 mg every other day) for those who need continued administration; if the PLT is ＞250×10^9/L, Eltrombopag should be discontinued and reinitiated until the PLT is ≤ 100×10^9/L.

Dosage for treatment failure during the study period

The dosage regimen after treatment failure during the study period is unlimited at the discretion of the investigator, with the recommended regimen as follows:
The rhTPO group may 1) combine the original investigational drug with other ITP agents, or 2) replace rhTPO with Eltrombopag, or 3) other;
The Eltrombopag group may 1) combine the original study drug with other ITP agents, or 2) replace Eltrombopag with rhTPO, or 3) other.

Concomitant therapy:
Subjects may receive hormone or immunosuppressive therapy as concomitant treatments for ITP at baseline, including but not limited to glucocorticoids, azathioprine, cyclosporine A, and danazol; hormones or immunosuppressants for ITP at baseline should maintain a stable therapeutic dose for at least the past month. During the treatment period, hormones or immunosuppressants may be tapered based on PLT (such as PLT rising to >50×10^9/L on two consecutive occasions or to >100×10^9/L) and patient's clinical manifestation.
Drugs beyond the investigational drugs may be administered at any time during the treatment period if the investigator determines that salvage therapy is necessary.

Prohibited drugs:
The following drugs are prohibited during the study (including but not limited to):
1) Other TPO-RAs (such as Herombopag, Romiplostim);
2) Platelet-stimulating traditional Chinese medicine.
During the treatment period of this study, no platelet boosting drugs other than the investigational drug in this study protocol shall be used for treatment, except for conversion treatment and salvage therapy after treatment failure.

Follow up duration:
Treatment period: D1 - D42
Follow-up period: D43 - D180

Criteria for dropout and withdrawal
The subjects are unwilling to continue participating in this study. Subjects drop out or are lost to follow-up, etc.; the investigator determines that there is an unacceptable risk for the subjects to continue participating in the study;
All subjects who withdraw from the study for adverse events or laboratory abnormalities must be followed up until their symptoms (test values) return to the state before the clinical study or to the extent that there are no medical problems. Additionally, the outcomes of this follow-up should be documented in the Case Report Form (CRF).

Elimination criteria:

1) Wrong treatment grouping;
2) Violation of the “concomitant medication” as specified in the protocol;
3) Failure to administer as prescribed in the protocol, which affects efficacy assessment;
4) Incomplete data which affects the assessment of efficacy and safety

Evaluation endpoints:
Primary endpoint: Median time to first PLT response ≥ 50 $\times 10^9$/L within 6-week treatment.
Secondary endpoints:
1) The proportion of patients with platelet count ⩾ 50 × 10^9/L at least once by week 6;
2) The proportion of patients who has an overall response or complete response at 1, 4, and 6 weeks;
3) Time to response and complete response;
4) The proportion of patients who responded at four or more of the last six visits at 4 or 6 months;
5) The duration of platelet count ⩾ 50 × 10^9/L;
6) The median time to treatment failure(TTF);
7) The proportion of patients with bleeding at week 1 to 6;
8) The proportion of patients with reduced or discontinued baseline concomitant treatment for ITP;
9) The proportion of patients requiring rescue therapy;
10) Adverse events.

BACKGROUND

Therapeutic Goals and Principles for Primary Immune Thrombocytopenia (ITP)

Primary immune thrombocytopenia (ITP) is an acquired autoimmune hemorrhagic disorder characterized by isolated decrease in peripheral blood PLT without clear triggering factors. ITP is classified into three stages based on the disease duration[1]: (1) Newly diagnosed ITP: patients within 3 months of diagnosis; (2) Persistent ITP: patients with ITP lasting between 3 and 12 months from diagnosis, including those not achieving spontaneous remission or those unable to maintain a response after stopping treatment; (3) Chronic ITP: patients with ITP lasting for more than 12 months.

The clinical manifestations of ITP vary greatly, including asymptomatic thrombocytopenia, skin and mucosal bleeding, severe visceral bleeding, and fatal intracranial hemorrhage. Platelet count is an independent risk factor for bleeding in patients with ITP [2]. Therefore, the treatments and interventions for adult patients with ITP depend on PLT and bleeding symptoms [3]. Multiple studies have shown that the risk and severity of bleeding in patients with ITP are associated with decreased platelet count. Generally, the lower the platelet count, the higher the risk of bleeding and the relatively more severe the bleeding[3]; previous studies have reported that when the PLT was < 20 × 10^9/L, the bleeding rate in adult patients with ITP was 86%; when the PLT was < 10 × 10^9/L, the bleeding rate increased to 94.8% [2]. Generally, patients with a PLT > 30 × 10^9/L rarely experience severe bleeding complications[4]. Consequently, both domestic and international guidelines for adult ITP use PLT < 30 × 10^9/L as a treatment indication[2] [3]. For ITP patients with a PLT < 30 × 10^9/L, it is necessary to quickly increase their PLTs to a safe level to reduce bleeding[5].

The pathogenesis of ITP is related to the loss of immune tolerance to platelet autoantigens, leading to accelerated platelet destruction and insufficient platelet production by megakaryocytes [1]. Clinical treatment of ITP usually focuses on reducing platelet destruction and increasing platelet production. According to the treatment principles in the *Chinese Guideline on the Diagnosis and Management of Adult Primary Immune Thrombocytopenia (version 2020)*, the major first-line treatment options for ITP are glucocorticoids and intravenous immunoglobulins (IVIg) [1]. Glucocorticoid treatment options include: (1) high-dose dexamethasone (HD-DXM) therapy at a dose of 40 mg/day × 4 days, which can be administered for two cycles; (2) prednisone at 1 mg/kg/day, which should be reduced as soon as possible after onset of effect and discontinued within 6 to 8 weeks. IVIg treatment options are mainly used for urgent treatment or patients who are intolerant to or have contraindications to glucocorticoids. Second-line treatment options are considered for patients who cannot maintain efficacy after glucocorticoid reduction and discontinuation.

Platelet-stimulating drugs including rhTPO and Eltrombopag are the first choice recommended by the guidelines for second-line therapy. According to the *Chinese Guideline on the Diagnosis and Management of Adult Primary Immune Thrombocytopenia (version 2020)*, platelet-stimulating drugs take effect within 1 to 2 weeks, with an effective rate of more than 60%. After discontinuation, the efficacy cannot be maintained, so individualized maintenance treatment is required [1]. The recommended dose of rhTPO is 300 U/kg/day subcutaneously for 14 days, with individualized maintenance for patients who respond. For patients who do not respond after 14 days of treatment, the drug should be discontinued (Grade A recommendation, Level Ib evidence) [1]. The recommended dose of Eltrombopag is 25 mg, once daily, orally administered under fasting conditions. If there is no response after 2 weeks of treatment, the dose should be increased to 50 mg/day (maximum dose of 75 mg/day), and individualized dose modification should be made to maintain the PLT $\geq 50 \times 10^9$/L; if there is still no response after maintenance treatment at the maximum dose for 2 to 4 weeks, the treatment should be discontinued[1].

Given that long-term use of glucocorticoids is associated with many side effects in patients, such as osteoporosis, inducing or aggravating infections, metabolic disorders, cardiovascular complications, inducing or aggravating peptic ulcers, and rebound after drug withdrawal, how to reduce or discontinue glucocorticoids as soon as possible is one of the important goals of improving ITP treatment options both domestically and internationally.

Regardless of the treatment options, the principle of all treatments is to elevate the PLT to a safety level and reduce bleeding events based on minimizing drug adverse reactions [1] [6]. As early as 2009, the International Working Group (IWG) proposed that the treatment goals for ITP with different disease durations vary slightly based on this treatment principle. For newly diagnosed ITP, the therapeutic goals include rapidly promoting the PLT to 50×10^9/L or more to prevent or stop bleeding, reducing treatment-related toxicity, and ensuring the quality of life. For persistent ITP or chronic ITP, it needs to maintain a stable PLT with safe drugs.

Review of Clinical Application of rhTPO in the Treatment of Adult ITP

Recombinant Human Thrombopoietin Injection (hereinafter referred to as rhTPO, trade name: TPIAO®) was approved by China's National Medical Products Administration (NMPA) for the treatment of adult ITP in 2010 [7]. In the *Chinese Guideline on the Diagnosis and Management of Adult Primary Immune Thrombocytopenia (version 2020)*, rhTPO is listed as a second-line and urgent treatment drug for adult ITP and has been gradually moved forward to the first-line treatment in clinical application [1] [8]. Real-world data has shown that rhTPO has demonstrated short and long-term efficacy and safety profiles for the treatment of adult ITP and can be used in the short or long term for the treatment of ITP [9] [10]. Due to its good safety profile, rhTPO is also recommended by the guideline for use in special populations such as pregnant women and children [1] [11]. rhTPO can be used in combination with various ITP therapeutic agents to form a variety of combination regimens, thereby meeting the individualized treatment needs of ITP patients [1] [8] [9] [12].

RhTPO can quickly increase the level of PLT. In the *Chinese Expert Consensus on Emergency Management of Adult Thrombocytopenia in China (version 2022)*[13], the onset times of effect of several first-line and second-line treatment drugs are compared. The time to response(TTR) of hormones (prednisone or equivalent) or hormone pulse regimens for ITP treatment is 2 to 5 days. In contrast, the TTR for rhTPO in treating ITP or chemotherapy-induced thrombocytopenia is 3 to 5 days, while the TTR for TPO-RA agents like Eltrombopag in treating ITP is generally 1 to 2 weeks.

The approved indication of rhTPO in China is primary ITP that is ineffective to glucocorticoid therapy. RhTPO is administered at 300 U/kg of body weight/day, once a day for 14 days[14]. The main adverse reactions are described as follows. The adverse reactions observed are rare. Pyrexia, muscle aches, and dizziness may occur occasionally, but generally do not require treatment and

will usually resolve spontaneously. Symptomatic treatment can be used for individual patients with obvious symptoms. The overall post-marketing incidence of adverse reactions for rhTPO was 1.27%, and no new symptoms of adverse reactions were identified outside of the package insert [15].

A Brief Analysis of the Need for Optimized rhTPO Regimen for the Treatment of ITP in Clinical Practice

As mentioned above, the package insert for rhTPO [7] describes the dosage regimen for ITP indication as 300 U/kg/day, once a day for 14 days. This dosage regimen originates from the registered clinical study in 2010 in which rhTPO was approved for ITP indication [16]. In this study, 140 patients with ITP who were ineffective to glucocorticoid treatment were randomly assigned to the treatment group (rhTPO + danazol) and the control group (danazol). The treatment group was treated with rhTPO at 300 U/kg/day subcutaneously in combination with danazol for 14 days, and the control group was treated with danazol alone. The results showed that the complete response rate and response rate (complete response + good response) in the treatment group were 38.4% and 60.3%, respectively, which were significantly higher than 7.9% ($p = 0.0003$) and 36.5% ($p = 0.0104$) in the control group. The complete response was defined as PLT increasing to $\geq 100 \times 10^9$/L; the good response was defined as PLT increasing to 50×10^9/L or increasing by more than 30×10^9/L relative to the baseline level; the response was defined as complete response plus good response.

With the continuous emergence of thrombopoietin receptor agonists (TPO-RAs) and the development for the treatment of ITP, it is necessary to modify the dosage of platelet-stimulating drugs based on PLT, which has gradually become a treatment trend for this class of drugs. For example, dose adjustment regimen based on PLT have been developed for Romiplostim and Eltrombopag. The initial dose of Romiplostim for ITP [17] is 1 μg/kg subcutaneously once a week. After that, the dose can be appropriately increased or decreased based on the PLT and symptoms (the dose is modified in 1-week increment, with a maximum dose of 10 μg/kg once a week). If the PLT is $< 50 \times 10^9$/L, the dose is increased by 1 μg/kg; if PLT is $\geq 50 \times 10^9$/L and $\leq 200 \times 10^9$/L, the effective dose is maintained; if PLT is $> 200 \times 10^9$/L and $< 400 \times 10^9$/L, the dose is reduced by 1 μg/kg; if PLT $\geq 400 \times 10^9$/L, the medication is suspended and then resumed after PLT $\leq 200 \times 10^9$/L. The initial dose of Eltrombopag for ITP [18] is 25 mg orally once daily for 2 weeks. After that, the dose of Eltrombopag is adjusted based on the PLT (the dose is adjusted in 2-week increment, with a maximum dose of 75 mg/day). If PLT is $\geq 50 \times 10^9$/L and $\leq 150 \times 10^9$/L, the effective dose is maintained; if PLT $> 150 \times 10^9$/L and $\leq 250 \times 10^9$/L, the dose is reduced to the adjacent lower dose (such as 50 mg/day reduced to 25 mg/day) or the dosage frequency is reduced (such as 25 mg/day reduced to 25 mg every other day); if PLT is $> 250 \times 10^9$/L, the medication is suspended and then resumed after PLT is $\leq 100 \times 10^9$/L.

Clearly, the current dosage regimen in the package insert of rhTPO limits the applicability of this drug in clinical practice for ITP to a certain extent. As the only marketed recombinant human thrombopoietin in the world and a national industrial product in China, it is necessary for rhTPO to be in line with the international academic frontiers by exploring and establishing an optimized regimen that is highly integrated with clinical practice for ITP and the dose can be adjusted based on PLT, as well as evaluating the rationality and clinical benefit of the optimized regimen through clinical studies to guide clinical practice.

Review of Head-to-Head Study of rhTPO versus Eltrombopag in the Treatment of Adult ITP

Professor Yu Hu et al. initiated a head-to-head study of rhTPO versus Eltrombopag in the treatment of ITP in 2018 (ClinicalTrials.gov Identifier: NCT03771378). The study was designed as a randomized, double-blind, double-dummy trial. Ninety six (96) pre-treated patients with ITP with disease duration of $\geq$ 6 months and baseline PLT $< 30 \times 10^9$/L were randomly assigned to receive Eltrombopag 25 mg/day or rhTPO 300 U/kg/day for 2 weeks at a ratio of 1:1. The

primary endpoint was overall response after 14 days, that is, the proportion of patients with a PLT ≥ 50 × 10^9/L [19].

The results showed that after 2 weeks of treatment, the overall response rate in the rhTPO group was significantly higher than that in the Eltrombopag group (75.00% vs. 43.75%, p = 0.003), and the complete response rate (the proportion of patients with PLT ≥ 100 × 10^9/L and no bleeding) was 64.58% and 25.00%, respectively ($p < 0.05$). In addition, on D9, D12, and D15, a higher proportion of patients in the rhTPO group had platelet counts that reached ⩾30 × 10^9/L and increased to more than twice baseline or reached ⩾50 × 10^9/L at least once. The incidence of adverse events in the two groups of patients in the study was similar. This study demonstrated that short-term treatment of ITP with rhTPO can bring platelets to safe levels more quickly than Eltrombopag, thereby reducing bleeding risk and helping patients quickly get rid of the bleeding risk.

STUDY OBJECTIVE

To evaluate the efficacy and safety of optimized rhTPO regimen versus Eltrombopag in pre-treated adult patients with ITP for 6 weeks.

STUDY DESIGN

Overall Design

This study aims to evaluate the efficacy and safety of optimized rhTPO regimen versus Eltrombopag in the treatment of pre-treated adult ITP patients. This is a randomized, open-label, active-controlled prospective study, using a superiority trial design to validate the efficacy and safety of optimized rhTPO regimen vs. conventional treatment with Eltrombopag, to evaluate the rationality and clinical benefits of optimized rhTPO regimen.

In this study, it is planned to enroll up to 175 pre-treated adult ITP patients with a disease duration ≥ 3 months. The patients will be randomly assigned at a 2:1 ratio and stratified based on the baseline PLT level to the rhTPO group (n = 117) and the Eltrombopag group (n = 58), and treated with drugs as per the protocol and observed for 6 weeks. Follow-up will be conducted on D60 (± 3), D120 (± 3), and D180 (± 3) after enrollment. In this study, efficacy, safety and rhTPO anti-drug antibody data will be collected from the subjects. The median time to response of rhTPO versus Eltrombopag for the treatment of pre-treated adult patients with ITP for 6 weeks will be primarily evaluated. The treatment response rate, the median time to treatment failure (TTF), the incidence of adverse events, and incidence of rhTPO anti-drug antibody will also be observed in both groups.

Rationale of Study Design

The major rationale of this study includes the mechanism of action for endogenous TPO, the mechanism of action for platelet-stimulating drugs, the clinical treatment advantages of rhTPO, and the basis for optimized rhTPO regimen. The details are as follows.

Mechanism of endogenous TPO regulating platelet production and its negative feedback regulation

Binding of TPO to the extracellular domain of the c-MPL receptor results in the formation of c-MPL homodimer, which can activate the tyrosine kinase JAK2. Activated JAK2 can initiate phosphorylation of tyrosine residues in the intracellular domain of the c-Mpl receptor, which in turn binds to signaling molecules with SH2 domains and promotes phosphorylation of signaling molecule tyrosine residues. Thus, a series of signaling pathways, such as tyrosine protein kinase/signal transducer and transcription activator (JAK/STAT), phosphatidylinositol-3-kinase and serine/threonine protein kinase (PI3K/Akt), and Ras/mitogen-activated protein kinase (Ras/MAPK) were initiated. Meanwhile, on the other hand, through the expression of cytokine signaling pathway inhibitors such as SOCS and phosphatases (PTEN, SHP1, SHIP1), the

reduction in c-Mpl expression on the cell surface leads to the reduction in TPO binding sites, signal shutdown, and prevention of unlimited proliferation of bone marrow hematopoietic progenitor cells and megakaryocytes [20].

Mechanism of action of platelet-stimulating drugs marketed in China

TPO analogues that mimic the physiological effects of TPO include rhTPO and TPO-RA in China. TPO analogues simulate the binding of signal molecules to receptors. The size and structure of the signaling molecule, the binding force with the receptor, and the site of binding to the receptor may affect the stereo conformational changes of the receptor's transmembrane domain; and the stereo conformational changes of the receptor's transmembrane domain will affect the quantity and quality of downstream signal pathways and the negative feedback regulation of signaling transduction [21]. RhTPO simulates the 3D binding mode of endogenous TPO and c-Mpl receptor, and this binding mode can maintain good efficacy and safety. The binding site and affinity of TPO-RA to c-Mpl receptor are different from that of endogenous TPO, and the signal transduction effects and clinical treatment manifestations induced by its binding are also different accordingly.

As a short peptide drug that mimics TPO, Romiplostim binds to the extracellular domain CRM1 of the c-Mpl receptor in a similar manner to TPO, but its binding affinity is about 25% of TPO. This means that Romiplostim needs to bind a larger number of c-Mpl receptors to achieve the same signaling pathway and intensity as TPO [22]. Early fundamental study performed by Professor Balduini et al. showed that Romiplostim has a relatively weak stimulation on hematopoietic stem/progenitor cells and megakaryocyte endoreduplication. It mainly induces megakaryocytes to avoid apoptosis and achieve proliferation by strongly stimulating Akt, and the MAPK signaling pathway is weak. Thus, Romiplostim stimulates the production of a large number of megakaryocytes, but the number of polyploid megakaryocytes is small and the formation of precursor platelets is reduced [23]. Small-molecule compounds (e.g., Eltrombopag) bind to the transmembrane domain (TMD) site of the c-Mpl receptor. This study showed that Eltrombopag can act on human c-Mpl, leading to the dimerization of c-Mpl and bringing the dimer into an active conformation. However, this process is affected by amino acid residues W515 and H499 in the transmembrane domain of the c-Mpl receptor [24]. In *Thrombopoietin Mimetics*, written by Professor Kuter in 2013, several attribute differences between TPO-RA and TPO were described. Compared with TPO, STAT5 and Akt signaling initiated by TPO-RA are weaker [21]. Professor Bussel et al. compared and evaluated the mechanism of action of TPO and TPO-RA, suggesting that the MAPK signaling pathway triggered by Eltrombopag is weak, and does not activate or weakly activates the PI3K/Akt pathway [20].

Clinical therapeutic advantages of rhTPO

As mentioned above, the binding sites and affinities of rhTPO and TPO-RA to c-Mpl receptors are different, and the signaling pathways and intensities they initiate are also different, resulting in different efficacy and safety in clinical practice. RhTPO is characterized by its rapid response and good safety profile.

Mechanism of rapid PLT elevation with rhTPO

In phase I clinical studies of rhTPO, Romiplostim, and Eltrombopag, changes in PLT after a single dose were observed. The PLT began to increase significantly on D5 after a single dose of rhTPO, and reached the peak between D10 and D14[25]. The PLT began to significantly increase approximately on D7 after a single dose of Romiplostim, and reached the peak between D12 and D16[26]. After a single dose of Eltrombopag, no increase in PLT was observed, and a dose-dependent increase in PLT was observed after 10 days of treatment [27].

In the registration clinical study of rhTPO, ITP subjects with a PLT $\leq 20 \times 10^9$/L were screened and assigned to the treatment group (rhTPO+danazol) and the control group (danazol alone) at a 1:1 ratio. The treatment group was given rhTPO 1.0 μg/kg (300 U/kg) once a day for 14 days.

About 25% of patients achieved complete response (PLT $\geq 100 \times 10^9$/L) after 14 days of treatment. The median time to response in treatment group was 7 days, while that in the control group was 10 days (Source: internal data on rhTPO).

In the registered clinical study of Eltrombopag in China, ITP subjects with a PLT $\leq 30 \times 10^9$/L were screened. The initial dose of Eltrombopag was 25 mg/day, and the dose of Eltrombopag was modified according to the PLT, with a maximum dose of 75 mg/day. The results of the study showed that the median time to response in patients was 23 days [28].

The development process of human megakaryocytes mainly includes the differentiation of hematopoietic stem cells into committed megakaryocytic progenitor cells, namely burst-forming-unit megakaryocyte (BFU-MK), the differentiation of BFU-MK into pre-primitive megakaryocytic cells, also known as colony-forming unit–megakaryocyte (CFU-MK), and megakaryocyte differentiation and maturation and platelet precursor formation [29]. An in vitro study showed that it takes 21 days for human hematopoietic stem cells to differentiate into BFU-MKs, and 12 days for them to differentiate into CFU-MKs [30]. The process of megakaryocytes from polyploidization and maturation to platelet release takes about 5 days [31].

The time to response for rhTPO is faster compared with Eltrombopag, which may be due to two reasons. One reason is that rhTPO is a subcutaneous injection, so the drug does not need to be absorbed by the stomach and intestines and enters the bloodstream quickly to take effect. Another reason is that rhTPO activates the MAPK signaling pathway within a short period of time to regulate the intranuclear mitosis of undamaged megakaryocytes in patients, and promotes megakaryocyte polyploidization and maturation to rapidly release platelets. At the same time, the binding of rhTPO to c-Mpl receptors activates pathways such as JAK-STAT and PI3K/AKT to promote the proliferation of megakaryocyte precursor cells, or protects megakaryocytes from apoptosis to increase the number of normal megakaryocytes, thereby supplementing the sustainable release of platelets and contributing to the maintenance of PLT in the long term. Thus, rhTPO rapidly promotes platelet production by increasing the volume of megakaryocytes in the short term, and maintains platelet production in the long term by protecting megakaryocyte lineage cells from apoptosis and increasing the number of megakaryocytes.

As mentioned above, the intensity of MAPK signaling pathway triggered by Eltrombopag is weak, and the PI3K/Akt pathway is not activated or weakly activated. Eltrombopag mainly promotes platelet production by promoting the proliferation of megakaryocyte progenitor cells and increasing the number of megakaryocytes [20]. The difference in the mechanism of action between rhTPO and Eltrombopag is clinically manifested as different rates of increase in PLT, that is, there are differences in the time to PLT $\geq 50 \times 10^9$/L. The rapid onset of effect of rhTPO can bring clinical benefits to patients with ITP.

Safety advantages of rhTPO

In the adverse reactions section of the current package insert of rhTPO (TPIAO®), the description of adverse reactions is as follows. The adverse drug reactions observed are rare. Pyrexia, muscle aches, and dizziness may occur occasionally, but generally do not require treatment and will usually resolve spontaneously. Symptomatic treatment can be used for individual patients with obvious symptoms. No serious adverse reactions were observed in the phase III clinical study of this product. In terms of post-marketing adverse reaction surveillance, the *Post-marketing study of recombinant human thrombopoietin injection for adverse drug reaction monitoring* (*Chinese Journal of New Drugs and Clinical Remedies*, 2015, Issue 8) collected information on a total of 6829 patients who had been treated with rhTPO from May 2008 to April 2014. The overall incidence of adverse reactions was 1.27%, and no new adverse reactions were identified that were not included in the package insert [15]. In addition, the periodic safety update reports (from May 2015 through December 2021) for rhTPO shows that symptoms of rhTPO-related adverse reactions involved a total of 14 organ-systems; the major adverse

reaction manifestations were pyrexia, fatigue, injection site reactions, rash, and pruritus.

The incidence of anti-drug antibodies is low during rhTPO treatment. In the phase I clinical study of rhTPO, no anti-drug antibodies were observed. In the phase II and phase III clinical studies, the incidence of anti-drug antibodies was 3.7% (3/81), all of which were low-titer anti-rhTPO non-neutralizing antibodies [32].

In the adverse reactions section of the package insert for Romiplostim for Injection (Romiplate®), the description of adverse reactions is as follows: clinical study conducted overseas showed the most common adverse reaction was headache, with an incidence of 35% in romiplostim treatment group. The adverse drug reactions observed are relatively rare, including recurrence of thrombocytopenia after treatment discontinuation, increased bone marrow reticulin, and thrombocythemia [17]. In the clinical studies conducted in China, a total of 45.7% of subjects in the Romiplostim group experienced adverse reactions during the trial. Adverse reactions with an incidence of ≥ 5% included joint pain (7.3%), and dizziness (6%). A total of 60.2% of subjects experienced adverse reactions after administration of Romiplostim. Treatment-related thrombotic events occurred in 2% (3/151) of subjects in the Romiplostim group. In the clinical studies conducted overseas, the incidence of anti-drug antibodies (ADAs) during treatment with Romiplostim in adults was 5.7% (60/1046). The incidence of ADAs to endogenous TPO during treatment with Romiplostim was 3.2% (33/1046). Of the patients who were positive for ADAs to Romiplostim or TPO, four (4) patients had ADAs that had neutralizing activity against Romiplostim, but these ADAs did not cross-react with endogenous TPO.

In the adverse reactions section of the package insert for Eltrombopag Olamine Tablets (Revolade®), the description of adverse reactions is as follows. Data from the clinical studies in ITP showed an overall incidence of adverse events in the safety analysis set was 79% (433/530), with the most common serious adverse reactions identified being hepatotoxicity and thrombosis/thrombotic events. The most common adverse reactions of all grades (occurred in at least 10% of patients) include headache, anemia, decreased appetite, insomnia, cough, nausea, diarrhea, alopecia, pruritus, myalgia, pyrexia, fatigue, influenza-like illness, weakness, chill and edema peripheral. Moreover, a black box warning was used to indicate the high risk of hepatotoxicity [18].

It should be noted that both Romiplostim and Eltrombopag have been associated with varying degrees of myelofibrosis risks in previous clinical studies.

Long-term Treatment with Romiplostim in Patients with Chronic Immune Thrombocytopenia: Safety and Efficacy, published by Professor David J. Kuter, et al. in 2013, showed that 292 patients received an average of 110 weeks of Romiplostim treatment; of the 38 patients who received bone marrow examinations, 11 patients experienced reticulin increased in 12 biopsies of the bone marrow [33]. Professor David J. Kuter et al. also observed that the degree of myelofibrosis caused by Romiplostim was related to the dose of Romiplostim in an animal study [34].

The EXTEND study of Eltrombopag was published in the *Blood* in 2016, titled "*Safety and Efficacy of Eltrombopag for Treatment of Chronic Immune Thrombocytopenia: Results of the Long-Term, Open-label EXTEND Study*". Long-term follow-up results showed that 147 of 299 patients were evaluated with reticulin/collagen testing, of which 48 had myelofibrosis grade 1 and 11 had myelofibrosis grade 2 [35]. Professor Başak Bostankolu Değirmenci et al. conducted a retrospective analysis of 27 patients who received Eltrombopag after allogeneic hematopoietic stem cell transplantation (alloHSCT). Of the 22 patients who received bone marrow aspiration, 12 were diagnosed with mild to moderate myelofibrosis. The grade of myelofibrosis was correlated with the time-to-treatment response ($p = 0.008$). The study suggested that long-term use of TPO-RAs may be considered as a potential cause of myelofibrosis in alloHCT recipients [36].

The cause of TPO-RA-induced myelofibrosis is unclear, and whether it is related to overstimulation of the proliferation of bone marrow hematopoietic progenitor cells is unknown.

In an article titled "*Literature Analysis of Adverse Reactions Caused by Thrombopoietin Receptor Agonists*" published in the *Chinese Journal of Drug Application and Monitoring* in February 2022, case reports of adverse drug reactions (ADRs) caused by Romiplostim and Eltrombopag were collected and statistically analyzed. The ADRs of these two drugs involved multiple systems/organs, mainly the circulatory system disorders (36.74%) and the hematological disorders (34.70%), and these ADRs may cause serious consequences once they occur [37].

Justification for optimized rhTPO regimen

This study optimized the dosage and frequency of rhTPO based on PLT. The justification for the optimized regimen mainly includes: 1) the dosage regimens as described in the package inserts for rhTPO and Eltrombopag; 2) the safety and efficacy of rhTPO at 600 U/kg/day; 3) the safety and efficacy of high dose rhTPO; 4) the safety and efficacy of rhTPO given by every other day. The rationale for administration is as follows.

Dosage regimens for rhTPO and Eltrombopag in their package inserts

For details, please refer to Section 2.3.

Safety and efficacy of rhTPO at a dose of 600 U/kg/day

In the phase I clinical studies, the pharmacodynamics, pharmacokinetics and tolerability of rhTPO were investigated in the range of 75 U/kg to 600 U/kg. The results showed that after a single dose of rhTPO at 150 U/kg, 300 U/kg and 600 U/kg, the PLT increased by 24%, 32% and 52%, respectively compared with the pre-dose level. RhTPO showed a dose-response relationship in increasing PLT, and the subjects in each group were well tolerated. No adverse events occurred in the 600 U/kg dose group, and the physical signs were stable, with no abnormal changes in serum biochemistry, urinalysis and hematological parameters (except for PLT) [38].

Safety and efficacy of high dose rhTPO

The efficacy and safety of rhTPO at high dose for treatment of ITP have been studied in the real world. At the 2022 academic conference organized by the International Society on Thrombosis and Haemostasis (ISTH), professor Zeping Zhou's team represented preliminary results comparing the efficacy and safety of rhTPO at doses of 15,000 U/day (n = 44) and 30,000 U/day (n = 61) for the treatment of ITP [39]. The results showed that the overall response rate in 105 patients was 82.9%, of which 29 were partial responders (27.6%) and 58 were complete responders (55.3%). The overall response rate in the 30,000 U/day group was significantly higher than that in the 15,000 U/day group (91.8% vs. 70.5%, $p = 0.004$). Compared with the 15,000 U/day group, the efficacy of the 30,000 U/day group on D7 and D14 was still more significant (38.6% vs. 73.8%, $p < 0.001$; 68.2% vs. 91.7%, $p < 0.01$), and a shorter time to response and longer duration of efficacy were observed in the 30,000 U/day group. The overall response rate in relapsed patients after resuming rhTPO was also better. The increase in rhTPO dose did not lead to an increase in the number of adverse events.

Safety and efficacy of rhTPO every other day

In an early clinical study, the pharmacodynamic and pharmacokinetic profiles of rhTPO were observed for daily or every other day regimen [40]. The results showed that the trough concentrations (C_{min}) in every other day regimen group and the daily regimen group reached the steady state level after 5 and 7 doses, respectively, and the trend of changes in the peak concentration (C_{max}) in the two groups was similar to that of C_{min}, and there was no significant difference in the pharmacokinetic parameters between the first and the last doses. The increase in

plasma concentration was positively correlated with the cumulative dosage administered, and there was no tendency for the drug to accumulate in body. Therefore, rhTPO with every other day regimen is as safe and effective as the daily regimen.

Professor Xiaowu Zhao et al. initiated a multicenter, randomized controlled study to explore the dosage regimen of rhTPO used for maintenance administration in the treatment of ITP [9]. The treatment group of this study used rhTPO in combination with prednisone, initially at 15,000 U/day for 14 days, followed by 15,000 U every other day for 90 days. The results showed that the PLT levels in the treatment group of rhTPO in combination with prednisone were significantly higher than those in the control group of prednisone alone on D8, D15, D22, and D30 of treatment. In addition, the early combination of rhTPO with hormones can not only quickly increase the PLT, but also have a long-lasting effect and reduce the dose of hormones. During the maintenance period of this study, rhTPO was administered every other day, which reduced the frequency of injections to some extent and provided convenience for patients.

Professor Yongqiang Zhao et al. optimized rhTPO regimen for ITP maintenance treatment [10]. For patients who responded after receiving rhTPO 300 U/kg/day for 2 weeks, maintenance treatment with reduced dose of rhTPO was continued for 12 weeks. The maintenance dosing regimen was rhTPO 300 U/kg with an initial dosing interval of every other day. PLT was monitored weekly and the rhTPO dosing interval was modified according to the patient's PLT. At Weeks 4, 8, and 12 in maintenance treatment period, the treatment response rates in the study were 92.6% (63/68), 82.7% (43/52), and 85.0% (34/40), respectively. The median PLTs of all treated patients fluctuated between 70×10^9/L and 124×10^9/L. The incidence of rhTPO-related adverse reactions was 7.7%, and the severity of all adverse reactions was mild. Therefore, maintenance treatment with a reduced dose of rhTPO at extended dosing intervals can maintain PLT at a safe level in most patients with ITP. It is necessary to further investigate and validate the extension of the dosing interval and, if appropriate, the dosing regimen of increasing the single dose in this study.

Sample Size

The study sample size is 175 subjects.

Based on previously published literature and clinical experience, it is estimated that the median time to first platelet $\geqslant 50 \times 10^9$/L in the rhTPO group is about 8 days, while the eltrombopag group is about 13 days. Group sequential trials with group sample sizes of 58 and 117 at the final look achieve above 80% power to detect a hazard ratio of 0.61 at the 0.05 significance level (two-sided alpha) using a two-sided log-rank test. We planned to conduct an interim analysis and use the Peto method to control the overall α (1st α 0.001, 2nd α 0.050).

Interim Analysis

In this study, the interim analysis is preplanned to be conducted after completion of enrollment of 105 evaluable subjects. Sample size will be re-estimated after the interim analysis.

STUDY POPULATION

Study population description: Pre-treated adults with immune thrombocytopenia (ITP).

Inclusion Criteria

Patients are required to meet all of the following inclusion criteria for participation in this study:

1) Male or female, aged ≥ 18 years;
2) Patients with at least 3 months history of ITP who have received treatment for ITP;
3) Baseline platelet count (PLT) < 30×10^9/L;
4) Patients receiving concomitant corticosteroids or immunosuppressants for ITP is eligible if

the doses are stable for at least 1 month;

5) Signed the informed consent form.

Exclusion Criteria

Patients who meet any of the following exclusion criteria must be excluded from the study:

1) Refractory ITP (refractory ITP is defined as ITP diagnosed on diagnostic reassessment in patients who have failed to response to first-line therapeutic drugs, platelet-stimulating drugs in second-line therapy, and rituximab, or who have failed to response to splenectomy/recurred after surgery);
2) History of arterial or venous thrombus or thrombophilia within the past year;
3) Having received any platelet increasing drug such as rhTPO, thrombopoietin receptor agonist (TPO-RA), etc. within 30 days;
4) Known to have poor efficacy with rhTPO or TPO-RAs;
5) Positive test results for hepatitis C virus antibody and human immunodeficiency virus antibody. Patients who are positive for hepatitis B virus surface antigen and have a quantitative test for hepatitis B virus DNA greater than 1000 cps/ml;
6) Creatinine and total bilirubin levels > 1.5 times the upper limit of normal, alanine aminotransferase and glutamic-oxaloacetic transferase levels > 3.0 times the upper limit of normal within the past 2 weeks;
7) History of malignant tumors;
8) Pregnant or breastfeeding women;
9) Any other situations deemed inappropriate by the investigator to participate in this study.

Criteria for Dropout and Withdrawal

If a subject does not wish to continue in the study, the informed consent may be withdrawn, and the investigator needs to be notified promptly if the subject decides to withdraw from the study. The reason for withdrawal from the study should be documented in the Case Report Form (CRF), including both the date and the specific reason for the withdrawal. Subjects who discontinue the study are required to complete all examinations outlined at the last visit (The investigator needs to confirm whether subjects who withdraw informed consent are willing to complete all examinations at the last visit). All subjects who withdraw from the study due to adverse events or laboratory abnormalities must be followed up until the symptoms (test values) return to the state before the clinical study or to the extent that there are no medical problems. Additionally, the outcomes of this follow-up should be documented in the Case Report Form (CRF). The criteria for a subject to discontinue the study also include dropping out or loss to follow-up. If a subject is lost to follow-up without withdrawing the informed consent, the investigator should make effort to contact the subject and document the contact attempts and other measures taken in the Case Report Form (CRF). If the investigator assesses that there is an unacceptable risk for the subject's continued participation in the study, the investigator has the right to terminate the subject's involvement in the clinical study.

Elimination Criteria

1) Wrong treatment grouping;
2) Violation of the Concomitant Medications as specified in the protocol;
3) Failure to administer as prescribed, which affects the assessment of drug efficacy;
4) Incomplete data affecting the assessment of efficacy and safety

INVESTIGATIONAL DRUG

1) Investigational Drug: Recombinant Human Thrombopoietin (rhTPO) Injection

Trade Name: TPIAO®

Manufacturer: Shenyang Sunshine Pharmaceutical Co., Ltd.

Strength/Approval No.:

7500 U/1 mL/vial, GYZZ S20050049

15000 U/1 mL/vial, GYZZ S20050048

Route of Administration: Subcutaneous administration

Storage: 2 to 8 °C, protected from light

2) Study Drug: Eltrombopag Olamine Tablets

Trade Name: Revolade®

Manufacturer: Novartis Pharma Schweiz AG

Strength/Approval No.:

Imported Drug Registration Certificate No

25 mg (calculated as $C_{25}H_{22}N_4O_4$) H20170387

50 mg (calculated as $C_{25}H_{22}N_4O_4$) H20170388

Route of Administration: oral

Storage: Store below 30 °C away from children

DOSAGE REGIMEN

The study comprises a 6-week randomized controlled treatment period and a follow-up period from D43 to D180.

General principles for drug administration during the randomized controlled observation period:

- During the observation period, the dose of study drugs is adjusted weekly according to the platelet count in both groups, with a maximum dose of 600 U/kg/day in the rhTPO group and 75 mg/day in the Eltrombopag group;
- The initial dose for both groups is determined based on baseline PLT, as described in the "Initial Dose" regimen for each group;
- After PLT reaches $\geq 50 \times 10^9$/L, the dose of the two groups is adjusted according to PLT level, as described in the "Dose Adjustment" regimen for each group;
- The dose of study drugs may be adjusted by the investigator temporarily or in advance, or alternatively used with adjacent doses when necessary, according to the treatment response and safety of patients.

During the 6-week observation period, the drug will be administered according to the following

regimen:

rhTPO group:

- Initial dose regimen: rhTPO will be administered at an initial dose of 300-600 U/kg by the investigator based on PLT together with the subject's treatment history; for patients with a PLT $\geq 20\times10^9$/L and $< 30\times10^9$/L, it should be administered at 300U/kg/day continuously; for those with a PLT $< 20\times10^9$/L, it should be administered at 600 U/kg/day continuously; for those with a PLT $< 50\times10^9$/L after 7 days of treatment, it should be administered at 600 U/kg/day continuously; rhTPO is administered subcutaneously;
- Dose adjustment regimen after PLT reaches $\geq 50\times10^9$/L: The rhTPO dose is adjusted weekly according to the platelet counts (PLT); if the PLT is $\geq 50\times10^9$/L and $\leq 150\times10^9$/L, rhTPO should be administered at 600 U/kg every other day; if the PLT is $> 150\times10^9$/L and $\leq 250\times10^9$/L, rhTPO should be administered at 450 U/kg every other day for those who need continued administration; if the PLT is $> 250\times10^9$/L, rhTPO should be discontinued, and reinitiated until the PLT is $\leqslant 100\times10^9$/L.

Eltrombopag group:

- Initial dose regimen: An initial dose of Eltrombopag at 25-50 mg/day will be administered by the investigator based on the baseline platelet count (PLT) and history of medical treatment for each subject. Subjects with $20\times10^9/L \leq PLT < 30\times10^9$/L and without active bleeding will be administered a continuous dose of 25 mg/day. Subjects with PLT $< 20\times10^9$/L or active bleeding will be administered a continuous dose of 50 mg/day. For subjects with $< 50\times10^9$ following continuous treatment for 7 days, the daily dose will be increased in 25 mg increments up to 75 mg/day. Eltrombopag is administered orally;
- Dose adjustment after PLT reaches $\geq 50\times10^9$/L: The Eltrombopag dose is adjusted on PLT levels. For subjects with $50\times10^9/L \leq PLT \leq 150 \text{ x } 10^9$/L, the effective dose will be maintained up to 75 mg/day. For subjects with $150\times10^9/L < PLT \leq 250\times10^9$/L, the dose may be reduced to an adjacent lower dose (e.g. 50 mg/day to 25 mg/day) or the frequency of administration may be reduced (e.g. 25 mg/day to 25 mg every other day) if continued treatment is required; if the PLT is $> 250\times10^9$/L, Eltrombopag should be discontinued, and reinitiated until the PLT is $\leqslant 100\times10^9$/L.

Dosage regimen during the follow-up period (D43 to D180):

The dosage regimen during the follow-up period is not restricted at the discretion of the investigator. It is recommended to continue the dosage regimen at the lowest dose of study drug that is adequate for maintaining PLT, preventing or minimizing bleeding, and/or in combination with ITP therapy.

Switching regimen after treatment failure:

The definition of treatment failure in this study is a platelet count $< 30\times10^9$/L after four weeks of treatment at the highest dose, a major bleeding event, or a change in therapy due to intolerable toxicities or bleeding (including minor bleeding).

Dosage regimen after treatment failure during the study period:

The dosage regimen after treatment failure during the study period is not restricted at the discretion of the investigator. Recommended regimens are as follows:

The rhTPO group may receive 1) the study drug in combination with another ITP agent or 2) a switch from rhTPO to Eltrombopag, or 3) other;

The Eltrombopag group may 1) the study drug in combination with another ITP agent, or 2) a switch from Eltrombopag to rhTPO, or 3) other.

Subjects who have failed treatment but are unwilling to remain under observation may receive rescue therapy.

Rescue therapy:

Rescue treatments, including glucocorticoids, intravenous globulin and platelet transfusion, were allowed during the treatment period, if the subject manifests a pronounced tendency towards bleeding or fails to respond to study drug.

PROHIBITED MEDICATIONS

The following drugs are prohibited during the study (including but not limited to):

1) Other TPO-RAs (such as Herombopag, Romiplostim);
2) Platelet-stimulating traditional Chinese medicine.

CONCOMITANT THERAPY

Subjects may receive hormones or immunosuppressive agents for treatment of baseline ITP, including but not limited to glucocorticoids, azathioprine, cyclosporine A, and danazol; such medications should be maintained at a stable therapeutic dose for at least the last month. During the treatment, dose reductions of hormones or immunosuppressants may be performed based on PLT (e.g., PLT increased to >50 × 10^9/L for two consecutive tests or to >100 × 10^9/L) and clinical signs of subjects.

Drugs other than the investigational drugs may be administered at any time during the study observation period if the investigator determines that salvage therapy is necessary.

SCHEDULE OF VISITS

Screening/Baseline

- Collection of medical history, including

1) Demographic data: Gender, age (date of birth), height, body weight, etc;
2) Complete medical history: History of pre-existing conditions and medical treatment, history of concomitant diseases and medical treatment, surgical history, etc;
3) Concomitant medications;
4) WHO bleeding score.

- Routine tests, including

1) Physical examination: General condition, skin, mucosa, lymph nodes, head, neck, chest, abdomen, spine and limbs, nervous system;
2) Laboratory tests (hematology, urinalysis, stool routine, blood biochemistry, coagulation function, and pregnancy test for women of childbearing potential);
3) Electrocardiogram (ECG)

- Pre-screening based on inclusion/exclusion criteria (as indicated in **5 Study Population**)

Treatment Period

1) Verification of inclusion/exclusion criteria;
2) Collection of vital signs;
3) Collection of dosage regimens in the study;
4) Frequency of visits for general parameters:
    a) Before PLT reaches ≥ 50×10^9/L:
        i. For subjects with PLT ⩾ 50×10^9/L within 3 weeks: Physical examination, hematology, WHO bleeding score, concomitant medications, platelet transfusion, adverse events, and clinical symptoms should be recorded every other day;
        ii. For subjects with PLT < 50×10^9/L after 3 weeks: Physical examination, hematology, WHO bleeding score, concomitant medications, platelet transfusion, adverse events, and clinical symptoms should be recorded twice a week;
    b) If PLT reaches ≥ 50×10^9/L: Physical examination, hematology, WHO bleeding score, concomitant medications, platelet transfusion, adverse events, and clinical symptoms should be recorded once a week;
5) Frequency of visits for other parameters (e.g. blood biochemistry, urinalysis, ECG):
    a) Blood biochemistry tests, once a week
    b) Urinalysis and ECG on D42 ± 1.

Follow-up Period

1) Collection of vital signs;
2) Collection of dosage regimens in the study;
3) The following items on D60 ± 3, D120 ± 3, and D180 ± 3 will be recorded: physical examination, hematology, blood biochemistry, urinalysis, ECG, WHO bleeding score, concomitant medications, platelet transfusion, adverse events, and clinical symptoms.

The schedule of visits is shown in Table 1-1 General Parameters (PLT < 50×10^9/L), Table 1-2 General Parameters (PLT ≥ 50×10^9/L), and Table 1-3 Other Parameters.

Table 1-1 General Parameters (PLT < 50×10^9/L)*

Test Item	Baseline (D-1 or D1 pre-dose)	Treatment Period										Follow-up Period		
		D3	D5	D7	D9	D11	D13	D15	D17	D19	Three visits a week until PLT ≥ 50×10^9/L	D60±3	D120 ±3	D180 ±3
Physical examination	X	X	X	X	X	X	X	X	X	X	X	X	X	X
Hematology	X	X	X	X	X	X	X	X	X	X	X	X	X	X
WHO bleeding score	X	X	X	X	X	X	X	X	X	X	X	X	X	X
Platelet transfusion record	X	X	X	X	X	X	X	X	X	X	X	X	X	X
Adverse event (AE)	X	X	X	X	X	X	X	X	X	X	X	X	X	X
Medication treatment	X	X	X	X	X	X	X	X	X	X	X	X	X	X
Concomitant medications	X	X	X	X	X	X	X	X	X	X	X	X	X	X

*For subjects with uncontrolled PLT within 3 weeks, the frequency of visits will be adjusted to 2 visits per week.

Table 1-2. General Parameters (PLT < 50×10^9/L)

Test Item	Baseline (D-1 or D1 pre-dose)	Treatment Period					Follow-up Period		
		+7 days after PLT reaches ≥ 50×10^9/L	+14 days after PLT reaches ≥ 50×10^9/L	+21 days after PLT reaches ≥ 50×10^9/L	+28 days after PLT reaches ≥ 50×10^9/L	+35 days after PLT reaches ≥ 50×10^9/L	D60 ± 3 days	D120 ± 3 days	D180 ± 3 days
Physical examination	X	X	X	X	X	X	X	X	X
Hematology	X	X	X	X	X	X	X	X	X
WHO bleeding score	X	X	X	X	X	X	X	X	X
Platelet transfusion record	X	X	X	X	X	X	X	X	X
Adverse event (AE)	X	X	X	X	X	X	X	X	X
Medication treatment	X	X	X	X	X	X	X	X	X
Concomitant medications	X	X	X	X	X	X	X	X	X

Table 1- 3. Other Parameters

Test Item	Baseline (D-1 or D1 pre-dose)	Treatment Period						Follow-up Period		
		D7	D15	D21±1	D28 ±1	D35 ±1	D42±1	D60±3	D120±3	D180±3
Blood biochemistry	X	X	X	X	X	X	X	X	X	X
Urinalysis	X						X	X	X	X
ECG	X						X	X	X	X
Urine pregnancy	X									

EVALUATION ENDPOINTS

Efficacy Endpoints

1) Median time to first platelet response: median time to first PLT ⩾ 50 ×10^9/L;
2) The proportion of patients with platelet count ⩾ 50 × 10^9/L at least once by week 6;
3) The proportion of patients who has an overall response or complete response at 1, 4, and 6 weeks; Overall response is defined as PLT ⩾ 30 × 10^9/L with an increase of at least twice the baseline count on two consecutive assessments (separated by ≥ 7 days) and the absence of bleeding; Complete response is defined as PLT ⩾ 100 × 10^9/L on two consecutive assessments (separated by ≥ 7 days) and the absence of bleeding;
4) Time to response and complete response;
5) The proportion of patients who responds at four or more of the last six visits at 4 or 6 months;
6) The duration of platelet count ⩾ 50 × 10^9/L;
7) The median time to treatment failure(TTF); Treatment Failure is defined as a platelet count < 30 × 10^9/L after four weeks of treatment at the highest dose, a major bleeding event, or a change in therapy due to intolerable toxicities or bleeding (including minor bleeding).
8) The proportion of patients with bleeding at week 1 to 6;
9) The proportion of patients with reduced or discontinued baseline concomitant treatment for ITP;
10) The proportion of patients requiring rescue therapy.

Safety Endpoints

- Adverse events

The observations for adverse events related to rhTPO include but are not limited to (refer to domestic Instructions for Use): fever, chill, general malaise, fatigue, knee joint pain, headache, dizziness, elevated blood pressure, rash, urticaria, somnolence, visual field defects, diarrhea, allergic reactions, injection site pain, etc.

The observations for adverse events related to Eltrombopag include but are not limited to (refer to domestic Instructions for Use): hepatotoxicity and thrombosis/thrombotic events, headache, anemia, loss of appetite, insomnia, cough, nausea, diarrhea, hair loss, itching, muscle pain, fever, fatigue, influenza like diseases, weakness, chill, and peripheral edema.

ADVERSE EVENTS AND SERIOUS ADVERSE EVENTS

All adverse events occurring from the date of signing ICF to the last visit will be recorded. Adverse events were graded and recorded according to NCI CTCAE 5.0

Adverse Event (AE)

Definition

Adverse events: Adverse medical events that occur in subjects after participating in clinical studies, which may not necessarily be related to the study treatment, include any newly occurring or worsening events in severity and frequency compared to baseline conditions, including abnormal results of diagnostic methods such as laboratory tests and physical examinations. Adverse events include severe adverse events (SAEs), adverse events (AEs), and laboratory findings.

Collection and record of AEs

The investigator shall record all suspected treatment-related adverse events from the date of signing the informed consent until the last visit with concise medical terminology.

Criteria of adverse event severity

The severity of adverse events is recorded according to Common Terminology Criteria for Adverse Events (NCI-CTCAE) Version 5.0 of National Cancer Institute, graded from 1 to 5.

Table 2: Common Terminology Criteria for Adverse Events (CTCAE) Version 5.0

Grade	Judgment criteria
Grade 1	Mild; asymptomatic or mild; only clinically or diagnostically seen; no treatment required
Grade 2	Moderate; requires minor, local or non-invasive treatment; age-appropriate instrumental limitation of activities of daily living*
Grade 3	Severe or medically significant but not immediately life-threatening; leading to hospitalization or prolonged hospitalization; disabled; restricted self-rational activities of daily living△
Grade 4	Life-threatening; urgent treatment required
Grade 5	Death related to AE

*Instrumental daily life activities: refer to cooking, buying clothes, using the phone, financial management, etc.
△Personal daily activities: refer to bathing, dressing and undressing, eating, washing, taking medication, etc., without being bedridden. Note to distinguish the severity and intensity of adverse events. Severe is used to describe the intensity, not necessarily SAE. For example, pain may be manifested as severe in intensity, but cannot be classified as SAE unless it meets SAE criteria.

Criteria for Determining the Relationship Between Adverse Events and Study Treatment

The correlation between adverse events and study treatment is analyzed by the investigators based on the principle of determining the correlation between adverse events and study treatment, which is classified into five categories: “related, possibly related, unlikely related, unrelated, and not evaluable”. AEs determined as "definitely related and possibly related" are counted as adverse reactions. Investigators must record adverse reactions using medical terminology.

Table 3: Criteria for determining the relationship between adverse events and study treatment

	Reasonable time relationship or not	Conform to the known types of adverse events or not	Possibility of adverse events occurrence due to other reasons or not
Related	Yes	Yes	Yes
Possibly related	Yes	Yes/No	Uncertain
Unlikely related	No	No	Uncertain
Not related	No	No	Yes
Not evaluable	Necessary information for evaluation not available		

Serious Adverse Event

Definition of serious adverse events

Serious adverse events may be defined as any of the following situations:

1) Death or life-threatening;
2) Requires hospitalization or prolonged hospitalization*;
3) Leading to obvious or persistent disability or functional impairment;
4) Leading to congenital abnormalities or defects;
5) A medically significant serious disease that endangers the health of patients and requires intervention through medical or surgical treatment to prevent any of the above outcomes.

*The following hospitalization treatments are often not considered serious adverse events:

1) Patients who have dropped out or withdrawn need hospitalization or prolonged hospitalization due to the progressive worsening disease;
2) Hospitalization for routine treatment or observation of indications involved in the trial, without deterioration of the condition;
3) The patient's hospitalization was planned before joining this trial, and the symptoms that were present before the trial did not worsen;
4) Only receiving treatment in the emergency or outpatient department, not meeting the definition of any serious adverse event mentioned above, and not requiring hospitalization;
5) Pregnancy itself is not a serious adverse event, while it should be reported on the Serious Adverse Event Form or Pregnancy Record Form, followed up and the results should be recorded, including natural abortion or voluntary termination of pregnancy, details of the baby's birth, whether congenital abnormalities and birth defects or not.

Reporting of Serious Adverse Events

If a subject experiences a grade 5 serious adverse event during the study (from the date of signing the informed consent to the last visit) that may be related to the study treatment, the investigators must fill out a serious adverse event report form and report it to the ethics committee of the study site within 24 hours from learning the event. The investigator must sign and date the report.

Medical Ethics Committee of Institute of Hematology & Blood Disease Hospital, Chinese Academy of Medical Sciences & Peking Union Medical College

Contact: Xue-ou Liu Tel.: 022-23909095; E-mail: ec@ihcams.ac.cn

Address: No.288 Nanjing Road, Tianjin, China

Pre-evaluation of Project Risks and its Mitigation

Recombinant human thrombopoietin (rhTPO) injection and Eltrombopag olamine tablet used in

this study are both indicated for primary immune thrombocytopenia (ITP) and have been recommended as treatment for adult ITP in the “Chinese Guidelines for Diagnosis and Treatment of Adult Primary Immune Thrombocytopenia (2020 Edition)”. The clinical symptoms and incidence of adverse events of the two products have been explained in the instructions, and there have been no reports of serious adverse events outside the scope of the instructions in ITP clinical practice since its marketing; The adverse events of the two products are predictable and controllable.

This study is a post-marketing IIT study, with the study population of “pre-treated adult with primary immune thrombocytopenia (ITP)”, which should be all within the indications of both products. Sufficient preliminary data and evidence are available for the study administration method; In summary, the technical risks in this study are highly controllable.

DATA MANAGEMENT

CRF is used for data collection in this study.

The contents of CRF will be entered into the database by a dedicated person assigned by the study site and reviewed by a dedicated person for the consistency between the database and CRF data.

The data manager checks the data in the database according to the clinical study protocol. For any questions, he/she fills out a query list, which shall be answered by the investigator. The query list should be properly kept.

The data manager is responsible for exporting data from the database for statistical analysis by statisticians.

STATISTICAL ANALYSIS

Statistical Analysis Set

Intent-to-Treat set (ITT): includes all randomized subject data.

Per-protocol set (PP): a subset of the full analysis set that does not include patients who affect the study results and clearly violate the protocol. The exclusion of the PP set should be determined before the database lock. This analysis set will be used for the analysis of the primary endpoint.

Safety set (SS): All subjects who receive at least 1 dose of study drug (rhTPO or Eltrombopag).

Statistical Analysis Method

Statistical description

The quantitative data is described statistically using the number of cases, mean, standard deviation(SD), median, minimum, and maximum. The statistical description of categorical variables is expressed using various patients’ cases and percentages. Unless otherwise specified, bilateral 95% is used as the width of all confidence intervals and the significance level of statistical tests.

Primary endpoint analysis

The primary endpoint for efficacy will be analyzed in the ITT and PP. The primary endpoint of this study is the median time to first PLT response $\geq 50\times10^9/L$ within 6 weeks treatment (median time to PLT $\geq 50 \times 10^9/L$). A stratified log-rank test on the median time to PLT $\geq 50\times10^9/L$ will be performed between the rhTPO group and the Eltrombopag group. Kaplan Meier method is used to plot survival curves, with a stratified Cox regression model for estimating the treatment effects .

14.2.3 Secondary endpoint analysis

Secondary endpoint analysis is performed using ITT. For quantitative data, t-test or rank-sum test are used to compare the inter group differences; For categorical data, Cochran-Mantel-Haenszel (CMH) test or Chi-Squared test are used to compare the inter group differences; For time to event analysis, Kaplan Meier method is used to plot survival curves, and stratified log-rank test for inter group comparison. A p-value of < 0.05 was considered statistically significant.

14.2.4 Safety endpoint analysis

Safety endpoints are analyzed in the SS. Adverse events are graded with CTCAE 5.0. The number of events, number of subjects with events, and incidence are summarized for adverse events, adverse reactions, serious adverse events and serious adverse reactions.

ETHICAL REVIEW

Ethical Requirements

This study is implemented according to the current Helsinki Declaration, relevant regulations, and ethical committee review opinions.

Before the start of the study, Investigators should obtain written approval opinions from relevant management units on the study protocol, informed consent form, subject recruitment procedures, and other written data to be provided to the subjects in accordance with regulations. During the study, for any additions or revisions to the study protocol, informed consent form, etc., written approval opinions from relevant management units should be obtained again in accordance with regulations.

Informed Consent

The Investigators or their designated representative will be responsible for explaining the study background, pharmacological characteristics of the study medical technology, study protocol, and the benefits and risks of participating in the study to all subjects (if applicable), their parents/legal guardians, or witnesses, and shall obtain written informed consent signed by the subjects themselves (if applicable), their parents/legal guardians, and the study physician before the subject enters the study (prior to screening examinations).

The informed consent form for this study includes the following contents: study purpose, study procedure, subjects obligations, foreseeable benefits and risks and inconveniences of participating in the study for the subjects; For any damage related to the study, the subjects may receive treatment; Access to study data and confidentiality of subject information.

Written approval shall be obtained for the informed consent form from the ethics committee of the study site in accordance with regulations, and the form shall be written in a language that is readable by the subjects (if applicable) and their parents/legal guardians. The subjects, their parents/legal guardians, investigators or their representatives who perform the informed consent process must sign and date the informed consent form. The original informed consent form should be kept by both the investigator and the subject. If new important data related to this study is found, the informed consent form must be revised in writing and submitted to the relevant management unit for approval before obtaining informed consent again.

For subjects who cannot participate in the informed consent process, written informed consent must be obtained from their parents/legal guardians, and all study procedures must be explained to them.

Confidentiality of Subjects

Investigators are responsibility for maintaining the anonymity of the subjects. Only uppercase letters, numbers, and/or codes can be used to identify subjects in the case report form or other documents, with subjects' names not used. Investigators must keep a subject screening and

enrollment form that records the subject's code, name, and home address. Investigators must strictly keep confidential any documents that can reveal the identity of the subjects.

STUDY MANAGEMENT

Training

Before the start of clinical study, investigators should receive training on the study protocol, read and understand the content in this clinical study protocol, unify the recording method and judgment criteria, and strictly follow the protocol.

Quality Control and Assurance

All observed results and abnormal findings in clinical study should be carefully verified and recorded in a timely manner to ensure the reliability of the data. The investigators input the protocol-specified information into the case report form, which will be verified by the monitor for the completeness and accuracy.

CONFIDENTIALITY AND PUBLICATION OF STUDY RESULTS

Investigators should keep confidential the information and data related to this study, and may not cite or publish relevant study results or materials without the consent of the study site.

REFERENCES

[1]. Hemostasis and Thrombosis Group, Hematology Society, Chinese Medical Association Chinese Guideline on the Diagnosis and Management of Adult Primary Immune Thrombocytopenia (version 2020). Chinese Journal of Hematology.2020;41(8):617-623.

[2]. M-L Piel-Julian, et al. Risk factors for bleeding, including platelet count threshold, in newly diagnosed immune thrombocytopenia adults. J Thromb Haemost. 2018 Sep; 16(9):1830-1842.

[3]. Li Jin, et al. Risk factors for bleeding in adult primary immune thrombocytopenia patients. Sichuan Medical Journal, 2019,40(12):1276-1280.

[4]. Quentin A Hill. Immune thrombocytopenia - in defence of the platelet count. Br J Haematol. 2018 Jul;182(1):128-130.

[5]. Francesco Rodeghiero, et al. Standardization of terminology, definitions and outcome criteria in immune thrombocytopenic purpura of adults and children: report from an international working group. Blood. 2009 Mar 12;113(11):2386-93.

[6]. Wang Ruting, et al. A physician-patient survey for primary immune thrombocytopenia: Chinese subgroup analysis of I-WISh International Survey. Chinese Journal of Hematology. 2021,42(5):369-375.

[7]. Shenyang Sunshine Pharmaceutical Co., Ltd., Recombinant Human Thrombopoietin Injection, Instructions for Use, Revised Edition on December 31, 2015.

[8]. YU Y, et al. High-dose dexamethasone plus recombinant human thrombopoietin vs high-dose dexamethasone alone as frontline treatment for newly diagnosed adult primary immune thrombocytopenia: A prospective, multicenter, randomized trial [J]. Am J Hematol. 2020, 95(12): 1542-52.

[9]. Xueli Jiao, et al. A Clinical Study on Treatment of Newly Diagnosed Immune Thrombocytopenia by Continuously Using Recombinant Human Thrombopoietin Combined with Prednisone [J]. Chinese Journal of Thrombosis and Hemostasis, 2017, 23(4): 545-9,52.

[10]. Huacong Cai, et al. A prospective study of the efficacy and safety of maintenance therapy with recombinant human thrombopoietin in patients with primary immune

thrombocytopenia: a multicenter study. Chinese Journal of Hematology.2017;38(5):379-383.

[11]. Working Group of Chinese Guideline for the Diagnosis and Treatment of Childhood Primary Immune Thrombocytopenia, et al. Adapted guideline for the diagnosis and treatment of primary immune thrombocytopenia for Chinese children (2021) [J]. Chinese Journal of Pediatrics. 2021, 59(10): 810-9.

[12]. Zhaoqi Yan, et al. Short-term efficacy and safety of rhTPO combined with traditional monotherapy regimen for the treatment of ITP: a Meta-analysis [J]. International Journal of Blood Transfusion and Hematology. 2018, 41(4): 277-84.

[13]. China Consensus Expert Group on Emergency Management of Thrombocytopenia in Adults. Expert consensus on emergency management of adult thrombocytopenia in China [J]. Chinese Journal of Emergency Medicine. 2022,31(2):161-168.

[14]. Shenyang Sunshine Pharmaceutical Ltd. Recombinant Human Thrombopoietin Injection Label Revised December 31, 2015.

[15]. Hou Ming, et al. Post-marketing study of recombinant human thrombopoietin injection for adverse drug reaction monitoring [J]. Chinese Journal of New Drugs and Clinical Remedies, 2015, 34(8): 642-6.

[16]. Wang Shujie, et al. The Multicenter Randomized-control Trial of Recombinant Humanized Thrombopietin Treatment in Patients with Idiopathic Thrombocytopenia Purpura [J]. Chinese Journal of Thrombosis and Hemostasis. 2010,16(4):149-153,157.

[17]. National Medical Products Administration. Romiplostim for Injection（JXSS2000008）- Label[J/OL] 2022,2022-05-20): https://www.cde.org.cn/main/xxgk/postmarketpage?acceptidCODE=99d4180bf 399db78f5d24ea396ee82fa.

[18]. National Medical Products Administration. Eltrombopag Olamine Tablets Label [J/OL] 2018,

https://www.cde.org.cn/main/xxgk/postmarketpage?acceptidCODE=2629ef0c1d4c51356 9a8 af4aa5ec7ed0.

[19]. MEI H, XU M, YUAN G, et al. A multicentre double-blind, double-dummy, randomised study of recombinant human thrombopoietin versus eltrombopag in the treatment of immune thrombocytopenia in Chinese adult patients [J]. Br J Haematol, 2021, 195(5): 781-9.

[20]. BUSSEL J, KULASEKARARAJ A, COOPER N, et al. Mechanisms and therapeutic prospects of thrombopoietin receptor agonists [J]. Semin Hematol, 2019, 56(4): 262-78.

[21]. KUTER D J. Chapter 59 – Thrombopoietin Mimetics, F, 2013 [C]

[22]. Varghese, L. N., Defour, J. P., Pecquet, C., & Constantinescu, S. N. (2017). The thrombopoietin receptor: Structural basis of traffic and activation by ligand, mutations, agonists, and mutated calreticulin. Frontiers in Endocrinology, 8(MAR), 1–13.

[23]. CURRAO M, BALDUINI C L, BALDUINI A. High doses of romiplostim induce proliferation and reduce proplatelet formation by human megakaryocytes [J]. PLoS One, 2013, 8(1): e54723.

[24]. VARGHESE L N, DEFOUR J P, PECQUET C, et al. The Thrombopoietin Receptor: Structural Basis of Traffic and Activation by Ligand, Mutations, Agonists, and Mutated Calreticulin [J]. Front Endocrinol (Lausanne), 2017, 8: 59.

[25]. Jiang Jieling, et al. Pharmacokinetics After a Single Subcutaneous Injection of Recombinant Human Thrombopoietin in Human [J]. The Chinese Journal of Clinical

Pharmacology. 2001, 17(4): 284-6.

[26]. WANG B, NICHOL J L, SULLIVAN J T. Pharmacodynamics and pharmacokinetics of AMG 531, a novel thrombopoietin receptor ligand [J]. Clin Pharmacol Ther, 2004, 76(6): 628-38.

[27]. ENKINS J M, WILLIAMS D, DENG Y, et al. Phase 1 clinical study of eltrombopag, an oral, nonpeptide thrombopoietin receptor agonist [J]. Blood, 2007, 109(11): 4739-41.

[28]. YANG R, LI J, JIN J, et al. Multicentre, randomised phase III study of the efficacy and safety of eltrombopag in Chinese patients with chronic immune thrombocytopenia [J]. Br J Haematol, 2017, 176(1): 101-10.

[29]. Hu Tao, Shi Xiaodong. Development and maturation of human megakaryocyte [J]. Chinese Journal of Pediatrics. 2005, 43(3):229-32.

[30]. BRIDDELL R A, BRANDT J E, STRANEVA J E, et al. Characterization of the human burst-forming unit-megakaryocyte [J]. Blood, 1989, 74(1): 145-51.

[31]. MACHLUS K R, ITALIANO J E, JR. The incredible journey: From megakaryocyte development to platelet formation [J]. J Cell Biol, 2013, 201(6): 785-96.

[32]. Hua Baolai, et al. Development of Recombinant Human Thrombopoietin Antibodies Following Multi Dosing, Subcutaneously Medication in Thrombocytopenic Patients [J]. Chinese Journal of Thrombosis and Hemostasis. 2005, 11(2): 59-61.

[33]. David J. Kuter, et al. Long-term treatment with romiplostim in patients with chronic immune thrombocytopenia: safety and efficacy. British Journal of Haematology, 2013,161:411–423.

[34]. David J Kuter. Evaluation of bone marrow reticulin formation in chronic immune thrombocytopenia patients treated with romiplostim. Blood. 2009;114:3748-3756.

[35]. Mansoor N Saleh, et al. Safety and efficacy of eltrombopag for treatment of chronic immune thrombocytopenia: results of the long-term, open-label EXTEND study. Blood. 2013;121(3):537-545.

[36]. Başak Bostankolu Değirmenci, et al. Eltrombopag may induce bone marrow fibrosis in allogeneic hematopoietic stem cell transplant recipients with prolonged thrombocytopenia. Leuk Res. 2022 Jul;118:106870.

[37]. Cao Shanshan, et al. Literature analysis of adverse drug reaction induced by thrombopoietin receptor agonists [J]. Chinese Journal of Drug Application and Monitoring. 2022, 19(1): 34-8.

[38]. Zhao Yongqiang, et al. Clinical tolerance test of recombinant human thrombopoietin [J]. Chinese Medical Journal. 2001, 81(24):1508-1511.

[39]. Wang X. Efficacy comparison and safety analysis of megadose recombinant human thrombopoietin(rhTPO) in the treatment of immune thrombocytopenia (ITP) . ISTH 2022 Congress.PB0338.

[40]. Zhu Tienan, et al. Pharmacokinetics after subcutaneous multi-dosing of recombinant human thrombopoietin in patients with thrombocytopenia[J]. The Chinese Journal of Clinical Pharmacology. 2004, 20(4):274-277.

[41]. Junyuan Qi, et al. Pharmacokinetics, Safety, and Pharmacodynamics of Romiplostim in Chinese Subjects with Immune Thrombocytopenia: A Phase I/II Trial. Clin Pharmacol Drug Dev. 2022 Mar;11(3):379-387

APPENDICES

Appendix 1: World Health Organization (WHO) Bleeding Scoring System

Due to the fact that the subjects are ITP patients, strict bleeding records must be kept, with the severity of bleeding described throughout the entire study duration. Mild to moderate bleeding includes no bleeding symptoms or only skin bleeding points and bruising, while severe bleeding symptoms include skin and mucosal bleeding as well as gastrointestinal, respiratory, urinary, and intracranial bleeding.

Grade	Bleeding type
Grade 1	Sparse and scattered skin petechiae and bruising<br>Nasal or oropharyngeal bleeding with a duration of < 30 min
Grade 2	Gastrointestinal, respiratory, musculoskeletal, or soft tissue bleeding that does not cause hemodynamic disorders and does not require transfusion of red blood cells within 24 h<br>Epistaxis or oropharyngeal bleeding with a duration of > 30 min<br>Symptomatic oral mucosal blisters<br>Scattered skin petechiae and bruising<br>Hematuria<br>Invasive procedures or abnormal bleeding at the surgical site<br>Non-menstrual vaginal bleeding<br>Serous cavity bleeding<br>Retinal hemorrhage without visual field defect
Grade 3	Bleeding requiring transfusion of red blood cells (especially within 24 h), while without severe hemodynamic disorders in the serosal cavity<br>Asymptomatic intracranial hemorrhage by CT scan
Grade 4	Retinal hemorrhage with visual field defect<br>Symptomatic non-fatal cerebral hemorrhage<br>Bleeding with hemodynamic disorders (hypotension, decrease in systolic or diastolic blood pressure > 30 mmHg)<br>Fatal bleeding for any reason

Appendix 2 Clinical Laboratory Examination Evaluation List

Blood test items	
Hematology	White blood cell classification count<br>Platelet count<br>Red blood cell count<br>Hemoglobin<br>Hematocrit<br>Neutrophil percentage<br>Lymphocyte percentage<br>Eosinophil percentage<br>Basophil percentage<br>Monocyte percentage<br>Basophil absolute value<br>Eosinophil absolute value<br>Neutrophil absolute value<br>Lymphocyte absolute value<br>Monocyte absolute value
Liver function	Alanine aminotransferase (ALT)<br>Aspartate aminotransferase (AST)<br>Alkaline phosphatase<br>Total bilirubin<br>Direct bilirubin
Renal Function	Blood urea nitrogen<br>Creatinine<br>Uric acid
Others	Hepatitis B DNA quantification
Urine examination items	
Urinalysis	Appearance<br>Color<br>pH<br>Specific gravity<br>Ketone body<br>Protein<br>Glucose<br>Nitrite<br>Urobilinogen<br>White blood cell<br>Red blood cell
Urine pregnancy test	hCG (diagnosis of pregnancy, female subjects with childbearing potential)

IIT2022037-EC-1 Version 1.1 Revision Details

Version of protocol	Date	Description of Amendment
1.0	August 1, 2022	
1.1	September 26, 2023	Revised several secondary study endpoints as follows:<br>4)The proportion of patients who responded at four or more of the last six visits at 3 or 6 months<br>**After versions**:<br>4) The proportion of patients who responded at four or more of the last six visits at 4 or 6 months<br>**Brief Rationale**:<br>We revised the 3-month response rate to 4-month response rate based on D120 visit.<br><br>Secondary endpoints:<br>11) Number of subjects who develop anti-rhTPO antibodies<br>**After versions**:<br>We have removed this secondary endpoint.<br>**Brief Rationale:**<br>Considering that ITP patients may intrinsically produce anti-TPO antibodies before study drug treatment and a reliable and accurate method for detecting anti-TPO or rhTPO antibodies has not yet been identified, we decided not to evaluate this secondary endpoint.<br><br>Treatment Failure is defined as a platelet count $\leqslant 20 \times 10^9$/L after four weeks of treatment at the highest dose, a major bleeding event, or a change in therapy due to intolerable toxicities or bleeding (including minor bleeding).<br>**After versions**:<br>Treatment Failure is defined as a platelet count $< 30 \times 10^9$/L after four weeks of treatment at the highest dose, a major bleeding event, or a change in therapy due to intolerable toxicities or bleeding (including minor bleeding).<br>**Brief Rationale:**<br>Treatment Failure definition in protocol version1.0 was based on one randomized controlled trial with romiplostim in immune thrombocytopenia(NCT00415532). Given that TE-ITP trial has enrolled baseline platelet count of 20~$<30 \times 10^9$/L and no response is usually defined as platelet of less than 30×10^9/L, we revised the platelet cut-off of treatment failure to be less than 30×10^9/L.<br><br>Deleted anti-drug antibodies against rhTPO test as follows:<br>10 SCHEDULE OF VISITS<br>10.1 Screening/Baseline<br>2) Laboratory tests (hematology, urinalysis, stool routine, blood biochemistry, coagulation function, and pregnancy test for women of childbearing potential) and baseline anti-drug antibodies against rhTPO;<br>**After versions**:<br>10 SCHEDULE OF VISITS<br>10.1 Screening/Baseline<br>2) Laboratory tests (hematology, urinalysis, stool routine, blood biochemistry, coagulation function, and pregnancy test for women of childbearing potential);

		**Brief Rationale:**<br>We decided not to evaluate anti-rhTPO antibody.<br><br>10.2 Treatment Period<br>5) Frequency of visits for other parameters (e.g. blood biochemistry, urinalysis, ECG):<br>a) Blood biochemistry tests, once a week;<br>b) Anti-drug antibodies tests against rhTPO on D15, D28 ± 1, and D42±1;<br>c) Urinalysis and ECG on D42±1.<br>**After versions**:<br>10.2 Treatment Period<br>5) Frequency of visits for other parameters (e.g. blood biochemistry, urinalysis, ECG):<br>a) Blood biochemistry tests, once a week;<br>b) Urinalysis and ECG on D42±1<br>**Brief Rationale:**<br>We decided not to evaluate anti-rhTPO antibody.<br><br>10.3 Follow-up Period<br>3) The following items on D60±3, D120±3, and D180± 3 will be recorded: physical examination, hematology, blood biochemistry, urinalysis, ECG, anti-drug antibodies against rhTPO, WHO bleeding score, concomitant medications, platelet transfusion, adverse events, and clinical symptoms.<br>**After versions**:<br>10.3 Follow-up Period<br>3) The following items on D60±3, D120±3, and D180± 3 will be recorded: physical examination, hematology, blood biochemistry, urinalysis, ECG, WHO bleeding score, concomitant medications, platelet transfusion, adverse events, and clinical symptoms.<br>**Brief Rationale:**<br>We decided not to evaluate anti-rhTPO antibody.<br><br>11.2 Safety Endpoints<br>● Adverse events<br>The observations for adverse events related to rhTPO include but are not limited to (refer to domestic Instructions for Use): fever, chill, general malaise, fatigue, knee joint pain, headache, dizziness, elevated blood pressure, rash, urticaria, somnolence, visual field defects, diarrhea, allergic reactions, injection site pain, etc. The incidence of rhTPO anti-drug antibody (ADA)/neutralizing antibody (NAB) during the rhTPO treatment is also observed.<br>**After versions**:<br>11.2 Safety Endpoints<br>● Adverse events<br>The observations for adverse events related to rhTPO include but are not limited to (refer to domestic Instructions for Use): fever, chill, general malaise, fatigue, knee joint pain, headache, dizziness, elevated blood pressure, rash, urticaria, somnolence, visual field defects, diarrhea, allergic reactions, injection site pain, etc.<br>**Brief Rationale:**<br>We decided not to evaluate anti-rhTPO antibody.<br><br>Table 1- 3. Other Parameters

Test Item	Baseline (D-1 or D1 pre-dose)	Treatment Period						Follow-up Period		
		D7	D15	D21±1	D28±1	D35±1	D42±1	D60±3	D120±3	D180±3
Blood biochemistry	X	X	X	X	X	X	X	X	X	X
Urinalysis	X						X	X	X	X
ECG	X						X	X	X	X
Urine pregnancy	X									
Anti-drug antibodies against rhTPO	X		X		X		X	X	X	X

After versions:

Test Item	Baseline (D-1 or D1 pre-dose)	Treatment Period						Follow-up Period		
		D7	D15	D21±1	D28±1	D35±1	D42±1	D60±3	D120±3	D180±3
Blood biochemistry	X	X	X	X	X	X	X	X	X	X
Urinalysis	X						X	X	X	X
ECG	X						X	X	X	X
Urine pregnancy	X									

Brief Rationale:

We decided not to evaluate anti-rhTPO antibody.

Appendix 2 Clinical Laboratory Examination Evaluation List

Anti-drug antibodies against TPO	Anti-TPO antibody (ADA)
	Anti-TPO neutralizing antibody (NAB)

After versions:

We removed anti-drug antibodies against TPO from Appendix 2 table.

Brief Rationale:

We decided not to evaluate anti-rhTPO antibody.
