# Supplementary material for: Dose-optimised recombinant human thrombopoietin versus eltrombopag in patients with immune thrombocytopenia: a multicenter, randomised controlled trial (The TE-ITP Study)
Source: eClinicalMedicine. 2025 Aug 21;87:103459. doi: 10.1016/j.eclinm.2025.103459 (PMC12396493; doi:10.1016/j.eclinm.2025.103459)
Supplement: Statistical Analysis Plan [file mmc3.pdf]

Statistical Analysis Plan

**Study Title**	Comparing the Efficacy and Safety of Optimized rhTPO Treatment Versus Eltrombopag Treatment in Previously Treated Primary Immune Thrombocytopenia Patients: A Multicenter Randomized Open-label Trial (TE-ITP)
**Protocol Number**	IIT2022037-EC-1
**SAP Version**	V1.1
**Date**	November 03, 2023

Table of Contents

Statistical Analysis Plan for a multicenter, open label, randomized controlled study of recombinant human thrombopoietin (rhTPO) versus eltrombopag in the treatment of adult ITP (TE-ITP) 3

1 Study Title 3

2 Study Objective 3

3 Study Design 3

3.1 Overall Study Design 3

3.2 Study procedures 3

4 Study Drug and Method of Administration 3

4.1 Study Drug 3

4.2 Dosing Regimen 4

5 Efficacy and Safety Evaluations 4

5.1 Efficacy Evaluation 4

5.1.1 Primary Efficacy Endpoint 4

5.1.2 Secondary Efficacy Endpoints 4

5.2 Safety Evaluation 5

6 Statistical Analysis 5

6.1 Sample size calculation 5

6.2 Analysis Sets 5

6.3 General Considerations 5

6.4 Patient disposition, demographics and other baseline characteristics . 6

6.5 Analysis of the primary objective 7

6.6 Analysis of the secondary objectives 7

6.7 Safety Analysis 8

Statistical Analysis Plan for a multicenter, open label, randomized controlled study of recombinant human thrombopoietin (rhTPO) versus eltrombopag in the treatment of adult ITP (TE-ITP)

This statistical analysis plan (SAP) is documented based on the protocol for " a multicenter, open label, randomized controlled study of recombinant human thrombopoietin (rhTPO) versus eltrombopag in the treatment of adult ITP (TE-ITP)" (protocol No.: IIT2022037-EC-1, version 1.1, dated 26 Sep 2023). The SAP will be finalized before the database is locked.

1 Study Title

A multicenter, open label, randomized controlled study of recombinant human thrombopoietin (rhTPO) versus eltrombopag in the treatment of adult ITP (TE-ITP).

2 Study Objective

To compare the efficacy and safety of rhTPO optimized administration with eltrombopag treatment for adult ITP treated for 6 weeks.

3 Study Design

3.1 Overall Study Design

This is a multi-center, randomized, open label, active-controlled clinical study.

3.2 Study procedures

The purpose of this study is to evaluate the efficacy and safety of the optimized administration of rhTPO compared to eltrombopag in the treatment of adult ITP. The study is a prospective randomized open-label, active controlled study that uses a superior efficacy trial design to validate the efficacy and safety of rhTPO optimized administration compared to conventional treatment with eltrombopag, in order to evaluate the rationality and clinical benefits of rhTPO optimized administration.

A total of 175 treated adult ITP patients with a disease duration of ≥ 3 months and platelet count of $<30\times 10^9$/L are selected for the study. They are randomly assigned into rhTPO group (n=117) and eltrombopag group (n=58) in a 2:1 ratio based on baseline platelet count levels, and treated with medication according to the study protocol and observed for 6 weeks; Follow up will be conducted on the 60 ± 3 days, 120 ± 3 days, and 180 ± 3 days after enrollment. Collect efficacy and safety data from subjects during the research process. The main purpose of this study is to compare the median time to achieve platelet count $\geq 50\times 10^9$/L for adult ITP patients treated with rhTPO or eltrombopag for 6 weeks, and to observe the treatment response rate, median time to treatment failure (TTF) and incidence of adverse events.

4 Study Drug and Method of Administration

4.1 Study Drug

1) Investigational drug: recombinant human thrombopoietin (rhTPO) injection

Product Name: TPIAO ®

Manufacturer: Shenyang Sunshine Pharmaceutical Co., Ltd

Specification/Approval Number:

7500U/1ml/vial, National Medical Products Approval No. S20050049

15000U/1ml/vial, National Medical Products Approval No. S20050048

Administration method: Subcutaneous injection

Storage: Store in the dark at 2-8 ℃

2) Research drug: Eltrombopag Olamine Tablets

Product Name: Revolade®

Manufacturer: Novartis Pharma Schweiz AG

Import drug registration certificate number

25mg (calculated as C25H22N4O4) H20170387

50mg (calculated as C25H22N4O4) H20170388

Administration method: Oral administration

Storage: Keep below 30 ° C and avoid contact with children

4.2 Dosing Regimen

The randomized controlled treatment period for the study is 6 weeks; The follow-up period is from day 43 to day 180.

During the treatment period, the initial dosage of rhTPO and eltrombopag was determined based on the baseline platelet count. After the platelet counts increase to 50×10^9/L or more, the dosage of rhTPO and eltrombopag will be adjusted on a weekly basis according to platelet response. The maximum dose of rhTPO is 600U/kg/day, and the maximum dose of eltrombopag is 75mg/day.

During the follow-up period, the treatment for anti-immune thrombocytopenia is not limited and determined by the investigator.

5 Efficacy and Safety Evaluations

5.1 Efficacy Evaluation

5.1.1 Primary Efficacy Endpoint

Time to the first platelet count $\geq50\times10^9$/L: Time from the start of treatment to the first time of achieving a platelet count $\geq50\times10^9$/L without salvage therapy during the first 6 weeks.

5.1.2 Secondary Efficacy Endpoints

Visits time points: baseline, and Weeks 1-6, Months 2, 4 and 6;

1) Overall Response at 1 week, 4 weeks, and 6 weeks

   Overall Response defined as platelet count $\geq30\times10^9$/L and at least a 2-fold increase of the baseline platelet count with absence of bleeding confirmed on two adjacent nominal visits at least 7 days apart.

2) Complete Response rates at 1 week, 4 weeks, and 6 weeks

   Complete Response: defined as a platelet count $\geq100\times10^9$/L with absence of bleeding confirmed on at least 2 separate occasions at least 7 days apart.

3) Response at month 4: Clinically meaningful platelet response of $\geq30\times10^9$/L and at least a 2-fold increase of the baseline count, for at least 4 out of 6 last visits until D120;

4) Response at month 6: Clinically meaningful platelet response of $\geq30\times10^9$/L and at least a 2-fold increase of the baseline count, for at least 4 out of 6 last visits until D180;

5) Cumulative number of days over the planned 6-Week treatment period with platelet counts of $\geq50\times10^9$/L.

6) Time to treatment failure over the planned 6-Week treatment period.

   Treatment failure is defined as:

- a platelet count $<30\times10^9$/L for 4 consecutive weeks at the highest dose and schedule
- a major bleeding event; or,
- a change in therapy due to an intolerable side effect or bleeding symptoms (including a minor bleeding event)

7) Changes from baseline in median of platelet count at each visit;
8) WHO bleeding events and severity by visit;
9) Usage of rescue therapy (Yes/No) during 6-Week treatment period;
10) Proportion of patients who reduced or interrupted baseline concomitant treatment for anti-immune thrombocytopenia during 6-Week treatment period.

5.2 Safety Evaluation

1) Occurrence of treatment-emergent adverse events (TEAEs).
2) Occurrence of treatment-related adverse events (TRAEs).

6 Statistical Analysis

6.1 Sample size calculation

Based on previously published literature and clinical experience, we estimated that the median time to respond in the rhTPO group is about 8 days, while the eltrombopag group is about 13 days. Group sequential trials with group sample sizes of 58 and 117 at the final look achieve above 80% power to detect a hazard ratio of 0.61 at the 0.05 significance level (two-sided alpha) using a two-sided Log-rank Test. We planned to conduct an interim analysis and use the Peto method to control the overall α (1st α 0.001, 2^{nd}α 0.050).

Sample Size Re-estimate

In this study, the interim analysis is preplanned to be conducted after completion of enrollment of 105 evaluable subjects. Sample size will be re-estimated after the interim analysis.

6.2 Analysis Sets

Descriptive statistics were used to summarize demographic and baseline characteristics and safety data. We included all patients randomly allocated treatment in the intention-to-treat population; the safety population was defined as all patients randomly allocated treatment who received at least one dose of study drug.

Safety Set (SS)

The SS will consist of all patients who receive at least 1 dose of study drug (rhTPO or Eltrombopag). Patients will be analyzed according to the treatment they received. This analysis set will be used in the analysis of demographic and safety data.

Randomized Set (RS)

The RS will include all enrolled study participants who were randomized. This is equivalent to the Intent-to-Treat (ITT) Set.

Per-protocol Set (PPS)

The PPS will consist of all patients in ITT set with no major protocol violations that may impact the analysis. Major protocol violations will be identified during the study according to the protocol deviation list and treatment compliance. Patients will be analyzed according to the treatment arm to which they were randomized. This analysis set will be used for the analysis of the primary endpoint.

6.3 General Considerations

Statistical analysis and generation of tables, figures, participant data listings, and statistical output will be performed using SAS® Version 9.4 or higher (SAS Institute, Cary, NC, USA).

Two-sided tests will be used for all statistical tests, with $P \leq 0.05$ considered as statistically significant.

Descriptive statistics will be displayed to provide an overview of the study results. For continuous variables, descriptive statistics will include number of participants with available measurements (n), mean, standard deviation (SD). For selected parameters, the median, 25th and 75th percentiles will be presented.

For categorical variables, the number and percentage of participants in each category will be presented. Unless otherwise noted, the denominator for percentages will be based on the number of participants included in the respective analysis set.

6.4 Patient disposition, demographics and other baseline characteristics

The Randomized Set (RS) will be used for all baseline and demographic summaries and listings unless otherwise specified.

Patient disposition

Disposition data will be summarized descriptively and listed. Summaries will include all reasons for treatment or study discontinuation as reported on the disposition eCRF pages. The flow diagram for patient disposition will be drawn.

Patient demographics and other baseline characteristics

Demographic variables will be summarized on the RS, by categories mentioned below using descriptive statistics, by treatment group and overall.

The last available assessment on or before the date of start of rhTPO or eltrombopag is taken as "baseline" assessment.

Continuous variables (including n, mean, SD):

- Age (years) -at the time of study entry.
- Weight (kg)
- Height (cm)
- BMI (kg/m^2) calculated as: BMI= Weight (kg)/ (Height (m))^2

Categorical variables (using frequency counts and percentages):

- Gender (Male, Female)

Baseline disease characteristics

Continuous variables (including n, median, Q1 and Q3):

- Time since first confirmed diagnosis of ITP (as collected in the eCRF)
- Platelet count ($\times 10^9$/L) (as collected in the eCRF)

Categorical variables (using frequency counts and percentages):

- Degree of thrombocytopenia (platelet count $<20\times10^9$/L or $\geq 20\times10^9$/L) (using data collected in the eCRF)
- Concomitant ITP medication at baseline (Hormones/immunosuppressant/No)
- Prior ITP treatments (corticosteroids, intravenous immunoglobulins (IVIg), thrombopoietic agents (rhTPO, TPO-RA, rhIL-11), anti-CD20 monoclonal antibody (rituximab), Danazol, immunosuppressant, Vinblastine/Vincristine, traditional Chinese medicine) (Yes/No)
- Splenectomy (Yes/No)
- PLT infusion (Yes/No)
- Baseline WHO Bleeding Score (0/1-4)

6.5 Analysis of the primary objective

The primary endpoint is the time to the first platelet count 50×10^9/L or more without salvage therapy during the first 6 weeks.

The primary endpoint will be analyzed with the stratified log-rank test and displayed using Kaplan-Meier curves. The same factors used for randomization will be used for stratification: baseline platelet count ($<20\times10^9$/L, $\geq 20\times10^9$/L).

Kaplan-Meier curves will be presented for the variables Time to the First Platelet Count $\geq 50\times 10^9$/L. The point estimate for the median number of days to response with 95% CIs will be given if estimable. Further a summary including the number and percent of censored subjects, the number of responders and total subjects by treatment groups will be given.

For the purpose of the analysis subjects with no response will be censored at the end of the study or at the date of withdrawal. Patients who had received rescue treatments were considered non-responders for the duration of rescue treatment and until platelet count decreased to < 50 x 109/after cessation of rescue treatment.

Analyses will be performed for the RS and repeated for the PPS.

The primary endpoint will be summarized using the median time achieving platelet response by each treatment group. The estimation of treatment effects will also be presented by Hazard Ratio (HR) estimated by stratified COX model in a 95% Confidential Interval (CI). The following subgroups of interest:

1) Sex (male, female)
2) Age (<overall median, ≥overall median)
3) Baseline platelet count ($<20\times10^9$/L, $\geq 20\times10^9$/L)
4) ITP duration (persistent ITP: ITP duration ≤ 1year, chronic ITP: ITP duration>1 year)
5) Baseline ITP concomitant medication (yes, no)
6) Baseline WHO bleeding score (0, ≥ 1)

6.6 Analysis of the secondary objectives

The number and percentage of subjects achieving Overall Response, Complete Response and Platelet Count $\geq 50\times10^9$/L will be summarized by treatment groups at each scheduled post-Baseline visit. The tabulation will also include an overall summary (across all visits) of the number and percentage of subjects achieving Overall Response, Complete Response and Platelet Count $\geq 50\times10^9$/L at any time during the study. The stratified Cochran-Mantel-Haenszel (CMH) test will apply for comparison of differences between groups.

Time to Response/Complete Response, and time to treatment failure will be analysis as the primary endpoint.

Summary statistics for the duration of platelet $\geq 50\times 10^9$/L within 6 weeks will be provided. Mean, SD, median, maximum, minimum, 25th percentile and 75th percentile will be provided. The Wilcoxon rank sum test will be applied for comparison of differences between groups.

Line plots for absolute change (median and mean) in platelet counts from baseline to different time points will be provided.

The proportion of patients who required rescue therapy and the proportion of patients who reduce or discontinue baseline ITP treatment within 6 weeks will be compared with the stratified CMH test.

The number and percentage of subjects with bleeding symptoms (WHO grades 1–4) will be summarized by treatment groups each week. A comparison will also made over the 6-week treatment period with a repeated measures model for binary data, with adjustment for the

randomization stratification variable and with use of generalized estimating equations method to estimate the regression model parameters.

6.7 Safety Analysis

- The analysis will be based on the actual data in the SS.
- Adverse events (AEs) will be assessed according to the Common Terminology Criteria for Adverse Events (CTCAE) version 5.0.
- Treatment-emergent adverse event (TEAE): defined as any AE that occurs or worsens after the first dose of the study drug.
- Treatment-emergent adverse event (TRAE): any adverse event that is assessed as "definitely", "probably", or "possibly" related to the study drug.
- Summarize the number, event and incidence rate of all TEAE, TRAE and SAE by grouping.

Statistical Analysis Plan Version 1.1 Revision Details

Version of SAP	Date	Description of Amendment
1.0	September 28, 2022	
1.1	November 03, 2023	Revised several secondary endpoints evaluation as follows:<br>Response at month 3: Clinically meaningful platelet response of $\geqslant 30 \times 10^9$/L and at least a 2-fold increase of the baseline count, for at least 4 out of 6 last visits until D120<br>**After versions**:<br>Response at month 4: Clinically meaningful platelet response of $\geq 30 \times 10^9$/L and at least a 2-fold increase of the baseline count, for at least 4 out of 6 last visits until D120<br>**Brief Rationale**:<br>Updated to align with Protocol V1.1<br><br>Treatment Failure is defined as a platelet count $\leqslant 20 \times 10^9$/L after four weeks of treatment at the highest dose, a major bleeding event, or a change in therapy due to intolerable toxicities or bleeding (including minor bleeding).<br>**After versions**:<br>Treatment Failure is defined as a platelet count $< 30 \times 10^9$/L after four weeks of treatment at the highest dose, a major bleeding event, or a change in therapy due to intolerable toxicities or bleeding (including minor bleeding).<br>**Brief Rationale:**<br>Updated to align with Protocol V1.1.<br><br>Revised Safety Evaluation as follows:<br>5.2 Safety Evaluation<br>1) Occurrence of treatment-emergent adverse events (TEAEs).<br>2) Occurrence of treatment-related adverse events (TRAEs).<br>3) Number of subjects who develop anti-rhTPO antibodies.<br>**After versions**:<br>5.2 Safety Evaluation<br>1) Occurrence of treatment-emergent adverse events (TEAEs).<br>2) Occurrence of treatment-related adverse events (TRAEs).<br>**Brief Rationale:**<br>Updated to align with Protocol V1.1.<br><br>Revised safety analysis as follows:<br>**6.7 Safety Analysis**<br>• Immunogenicity Analysis: numbers and percentage of subjects with positive anti-drug antibody (ADA) and neutralizing antibody (NAb) results at each planned visit are summarized. The analysis will be based on the actual data in the SS.<br>**After versions**:<br>We removed the Immunogenicity Analysis.<br>**Brief Rationale:**<br>Updated to align with Protocol V1.1.
